# CircCCNB1 silencing acting as a miR-106b-5p sponge inhibited GPM6A expression to promote HCC progression by enhancing DYNC1I1 expression and activating the AKT/ERK signaling pathway

**DOI:** 10.7150/ijbs.66915

**Published:** 2022-01-01

**Authors:** Yan-ming Liu, Yue Cao, Ping-sen Zhao, Liang-yin Wu, Ya-min Lu, Yu-long Wang, Jia-feng Zhao, Xin-guang Liu

**Affiliations:** 1 Guangdong Provincial Key Laboratory of Medical Molecular Diagnostics, Institute of Aging Research, Guangdong Medical University, Dongguan, Guangdong, China.; 2 Department of Clinical Laboratory, YueBei People's Hospital, Shaoguan, Guangdong, China.; 3 The Third Clinical Medical College, Guangzhou University of Chinese Medicine, Guangzhou, Guangdong, China.; 4 Department of Medical Technology, Medical College of Shaoguan University, Shaogguan, Guangdong, China.; 5 Department of Anesthesiology, YueBei People's Hospital, Shaoguan, Guangdong, China.; 6 Department of Hepatobiliary Surgery, YueBei People's Hospital, Shaoguan, Guangdong, China.

**Keywords:** Circular RNA, hepatocellular carcinoma, circCCNB1, miR-106b-5p, GPM6A, DYNC1I1, AKT/ERK signal pathway

## Abstract

**Background:** Circular RNAs (circRNAs), which generally act as microRNA (miRNA) sponges to competitively regulate the downstream target genes of miRNA, play an essential role in cancer biology. However, few studies have been reported on the role of circRNA based competitive endogenous RNA (ceRNA) network in hepatocellular carcinoma (HCC). Herein, we aimed to screen and establish the circRNA/miRNA/mRNA networks related to the prognosis and progression of HCC and further explore the underlying mechanisms of tumorigenesis.

**Methods:** GEO datasets GSE97332, GSE108724, and GSE101728 were utilized to screen the differentially expressed circRNAs (DE-circRNAs), DE-miRNAs, and DEmRNAs between HCC and matched para-carcinoma tissues. After six RNA-RNA predictions and five intersections between DE-RNAs and predicted RNAs, the survival-related RNAs were screened by the ENCORI analysis tool. The ceRNA networks were constructed using Cytoscape software, based on two models of up-regulated circRNA/down-regulated miRNA/up-regulated mRNA and down-regulated circRNA/up-regulated miRNA/down-regulated mRNA. The qRT-PCR assay was utilized for detecting the RNA expression levels in HCC cells and tissues. The apoptosis, Edu, wound healing, and transwell assays were performed to evaluate the effect of miR-106b-5p productions on the proliferation, invasion, and metastasis of HCC cells. In addition, the clone formation, cell cycle, and nude mice xenograft tumor assays were used to investigate the influence of hsa_circ_0001495 (circCCNB1) silencing and overexpression on the proliferation of HCC cells *in vitro and in vivo*. Furthermore, the mechanism of downstream gene DYNC1I1 and AKT/ERK signaling pathway via the circCCNB1/miR-106b-5p/GPM6A network in regulating the cell cycle was also explored.

**Results:** Twenty DE-circRNAs with a genomic length less than 2000bp, 11 survival-related DE-miRNAs, and 61 survival-related DE-mRNAs were screened out and used to construct five HCC related ceRNA networks. Then, the circCCNB1/miR-106b-5p/GPM6A network was randomly selected for subsequent experimental verification and mechanism exploration at *in vitro and in vivo* levels. The expression of circCCNB1 and GPM6A were significantly down-regulated in HCC cells and cancer tissues, while miR-106b-5p expression was up-regulated. After transfections, miR-106b-5p mimics notably enhanced the proliferation, invasion, and metastasis of HCC cells, while the opposite was seen with miR-105b-5p inhibitor. In addition, circCCNB1 silencing promoted the clone formation ability, the cell cycle G1-S transition, and the growth of xenograft tumors of HCC cells via GPM6A downregulation. Subsequently, under-expression of GPM6A increased DYNC1I1 expression and activated the phosphorylation of the AKT/ERK pathway to regulate the HCC cell cycle.

**Conclusions:** We demonstrated that circCCNB1 silencing promoted cell proliferation and metastasis of HCC cells by weakening sponging of oncogenic miR-106b-5p to induce GPM6A underexpression. DYNC1I1 gene expression was up-regulated and further led to activation of the AKT/ERK signaling pathway.

## Introduction

Hepatocellular carcinoma (HCC), which can be caused by chronic viral hepatitis (hepatitis B and C), liver cirrhosis, alcoholic liver disease, aflatoxin, etc., is a malignancy with a high worldwide prevalence, ranking second among cancer-related causes of death 1. Screening and therapeutic approaches have been implemented not only for early but also for advanced HCC stages. Nonetheless, current morbidity and mortality rates are still rising 2. Given the poor performance of current liver cancer markers used for early screening, surveillance, and diagnosis of HCC, treatment modalities including surgery, radiotherapy, and chemotherapy are not indicated for most HCC patients when they are first diagnosed 3. Accordingly, precise biomarkers are needed at the clinical level to improve the screening and detection of HCC at early stages and improve patient prognosis.

Due to the rapid development of high-throughput sequencing technology, a plethora of circular RNAs (circRNA) have been discovered 4. CircRNAs, with excellent specificity for cells, tissues, and disease stages in several kinds of tumors, have more stable characteristics than linear RNA due to their covalently closed structures 5. microRNA (miRNA), with a genomic length of 18-22 bp, mainly has down-regulated effects on its target genes via direct binding to the seed region of 3'-UTR of the target genes 6. Importantly, circRNAs with miRNA recognition element (MRE) can directly bind to miRNAs to indirectly protect downstream target mRNAs of miRNAs 7. Prior studies have revealed that a small group of circRNAs plays an essential role in the proliferation and metastasis of HCC cells via the circRNA/miRNA/mRNA ceRNA network 5. circMTO1 promotes p21 overexpression to weaken HCC progression by acting as the sponge of oncogenic miR-9 and is considered a novel early diagnosis, treatment, and prognosis predictor for HCC patients 8. Due to the unsatisfactory early diagnosis rates and patient outcomes and the vital role of the ceRNA network in HCC, screening and constructing functional circRNA/miRNA/mRNA ceRNAs networks is paramount to improve our understanding of HCC and develop new diagnostic and therapeutic measures.

In this study, we screened HCC survival-related differentially expressed circRNAs (DE-circRNAs)- miRNAs (DE-miRNAs)-mRNAs (DE-mRNAs) with RNA-RNA interaction using three GEO datasets and bioinformatics analysis tools and successfully constructed ceRNA networks. Then, we explored the expression and mechanism of the hsa_circ_0001495 (circCCNB1)/miR-106b-5p/GPM6A ceRNA network *in vitro* and *in vivo*. Importantly, we demonstrated that circCCNB1 silencing could promote down-regulation of the targeted GPM6A by miR-106b-5p induced reduction of miR-106b-5p sponging, which subsequently up-regulated DYNC1I1 to activate the AKT and ERK signaling pathways and enhance the proliferation and metastasis of HCC cells.

## Materials and methods

### Construction and function annotation of the ceRNA network

To explore the role of the circRNA/miRNA/mRNA ceRNA network in HCC pathogenesis, three HCC related GEO (https://www.ncbi.nlm.nih.gov/geo/) datasets including GSE97332 (circRNA) 8, GSE108724 (miRNA) 9, and GSE101728 (mRNA) 9 were selected and utilized for ceRNA construction. The GEO2R web tool 10, which can identify differential expressed genes between two or more datasets based on its built-in GEOquery and R package “limma”, was used for screening the differently expressed RNAs according to the cut-off criterion *P*-value<0.05 and |log_2_ fold change (log_2_FC)| > 1. The Encyclopedia of RNA Interactomes (ENCORI, http://starbase.sysu.edu.cn/index.php), an open-source platform that can identify more than 1.1 million miRNA-ncRNA and 2.5 million miRNA-mRNA, was applied for the prediction of miRNA-circRNA and miRNA-mRNA interactions, survival, differential expression, and co-expression in HCC 11. The UALCAN (http://ualcan.path.uab.edu/), a comprehensive, user-friendly, and interactive portal for the cancer OMICS analysis, was used for executing the Kaplan-Meier survival analysis for miRNAs and mRNAs in HCC 12. The baselines of circRNAs were derived from the circBase website (http://circbase.org/) 13 The Microsoft Access 2016 software was used to perform the sorting and intersection of expressed RNAs. The RNA expression heatmaps were drawn using the “pheatmap” R package. The Database for Annotation, Visualization, and Integrated Discovery (DAVID; https://david.ncifcrf.gov) (version 6.8) 14 offers functional annotation tools for understanding the biological meaning behind a large list of genes. It was used to perform the Gene Ontology (GO) terms and Kyoto Encyclopedia of Genes and Genomes (KEGG) pathway analysis. Cytoscape software (version 3.7.2), which can visualize complex networks between genes, was applied to visualize and establish the ceRNA regulatory networks composed of circRNAs, miRNAs, and mRNA 15.

### Silencing and overexpression of circCCNB1

The lentivirus vectors or negative control vectors of circCCNB1 silencing expression (si-circCCNB1, GIDL0269186) and overexpression (oe-circCCNB1, GOSL0269185) were designed and constructed by GeneChem company (Shanghai, China). HCC cells, infected with lentivirus, were screened continuously for two weeks using 2 μg/mL puromycin. After circCCNB1 silencing and overexpression efficacy verification, the lentivirus-infected HCC cells were used for the next steps of experiments.

### Real-time quantitative reverse transcription PCR (qRT-PCR)

Total RNA from HCC cells and tissues were extracted using the total RNA extraction kit (R4107; GBCBIO, Guangzhou, China). Then, RNA concentration was measured by Nanodrop. The Ribo SCRIPT^TM^ Reverse Transcription Kit and stem-loop primers (C11027-2/T1701-2, Ribobio) were used for the reverse transcription reaction of miRNAs. The Transcriptor First Strand cDNA synthesis Kit (0489703000; Roche) was applied for the reverse transcription reaction of circRNAS and mRNAs. Finally, qRT-PCR of circRNAs, miRNAs, and mRNAs was performed using the LightCycle 480 SYBR Green I Master Kit (04707516001; Roche) on the instrument of Lightcycler 480^®^ II with GAPDH and U6 as the internal control. The 2^-ΔΔCT^ method was applied to determine the relative expression level of RNAs. All the primers of RNAs were listed in Table S1.

### The analysis of cell cycle, apoptosis, and EdU

HCC cells (2 × 10^5^ cells/well) were plated in 24-well plates and growth to 95% confluence for harvesting. The cell cycle detection kit (C1052;), Annexin V-FITC cell apoptosis detection kit (C1062M), and EdU-488 cell proliferation detection kit (C0071S), purchased from Beyotime Company (Shanghai, China), were used for the analysis of cell cycle, apoptosis, and proliferation on the instrument of FACS ARIA II (BD Biosciences; USA) according to the manufacturer's instructions.

### Plate colony formation assay

HCC cells (200 cells/well) were plated in six-well plates and cultured for two weeks with 5% CO_2_ at 37 °C after being fixed and stained with crystal violet, the numbers of colonies per well were imaged and counted.

### Dual-luciferase report assay

Dual-Luciferase reporter vector with the wild type (WT) or mutant type (MUT) of circCCNB1 complete sequence or 3'UTR of GPM6A and their negative control (NC), purchased from Ribobio company (SA20210222012 for circCCNB1, SA20210222012 for GPM6A), were constructed based on the pmiR-RB-Report vector. HCC cells (10^4^ cells/well) were plated in 24-wells plates and continuously cultured for 24h to 60% confluence. The constructed luciferase vector and miR-106b-5p mimics or NC were transfected into HCC cells with the Lipo6000 transfection reagent (C0526; Beyotime). After 48 h of incubation, the relative light unit (RLU), positively correlated with activities of firefly luciferase (FL) and Renilla luciferase (RL), was observed using a dual-luciferase reporter kit (RG027; Beyotime) on the fluorescence spectrophotometer (FLUOROSKN FL, Thermo, USA). The RLU ratio of FL/RL was calculated for statistical analysis.

### Fluorescence *in situ* hybridization (FISH)

To explore the co-localization of circCCNB1 and miR-106b-5p, the circCCNB1 probe for FISH was 5'-biotin-ACAATTATTCCATTCACCAT, and the miR-106b-5p probe for FISH was 5'-digoxin-ATCTGCACTGTCAGCACTTTA were synthesized by Sangon Biotech (Shanghai, China). The Cy3-conjugated Streptavidin (D110514; Sangon), HRP-labeled rabbit anti-digoxigenin antibody (49620; Cell signaling, USA), iFluor 488^TM^ PSA^TM^ imaging kit with goat anti-rabbit IgG (AAT-45205; AAT Bioquest, USA), and Ribo^TM^ fluorescent *in situ* hybridization kit (C10910; Ribobio) were used for the fluorescence signal amplification according to the manufacturer's instruction manual. HCC cells (5 × 10^4^ cells/well) were plated on the round coverslip in advance in a 24-wells plate. After incubation for 24 hours, fixation, permeabilization, blocking, elimination of endogenous peroxidase, hybridization, and antibody incubation were performed on the HCC cells. Finally, the cells were incubated with Hoechst for 10 min. Images were captured by an Olympus IX83 confocal laser imaging system (Olympus, Japan).

### circRNAS *in vivo* precipitation (circRIP)

Biotin-labeled circCCNB1 probe (lnc40024707) and its control probe (lnc40024708) were designed and purchased from Ribobio company. The circRIP assay was performed according to the protocol previously reported with minor adjustments 16. CircCCNB1-overexpression-HCC cells (2.5 × 10^6^ cells/well) were plated in a 10cm cell culture dish and cultured for 24 h to 95% confluence. Then, the HCC cells were fixed by 1% formaldehyde for 10 minutes, lysed, and sonicated for ten cycles (parameters: high power, 30s ON, and 30s OFF). After centrifugation, 50 µl of the supernatant was defined as an input, and the remaining part was incubated with circCCNB1 probe or probe NC (final probe concentration: 400nM/L) and streptavidin dynabeads (M-280, Invitrogen, USA) overnight at 37 °C. The following day, the mixture of M-280 dynabeads-circCCNB1 probes-circCCNB1 was purified by magnetic separation and reversed the formaldehyde cross-linking. Finally, the above mixture was used for miRNA extraction and qRT-PCR detection.

### Western blotting (WB) and immunohistochemistry (IHC) assay

For the WB assay 6, HCC cells (1 × 10^5^ cells/well) were seeded in 6-wells plates and cultured for 24 h. After transfection and growth to 95% confluence, the cells were lysed, harvested, and performed protein qualification. The fresh HCC carcinoma tissues and para-carcinoma tissues were fixed with 4% paraformaldehyde, embedded in paraffin, and sliced for the IHC assay 17. The primary antibodies and their diluted working solutions were listed as follows: anti-GPM6A (Ab230356; Abcam, UK, 1:1000 for WB, and 1:200 for IHC), anti-β-actin (Ab8227; Abcam, UK, 1:1000 for WB), Anti- Cytoplasmic dynein 1 intermediate chain 1 (DYNC1I1, ab169758; Abcam, 1:1000 for WB), anti-AKT (AA326; Beyotime, 1:1000 for WB), anti-Phospho-Akt (AA329; Beyotime, 1:1000 for WB), anti-ERK1/2 (AF1051; Beyotime, 1:1000 for WB), anti-Phospho-Erk1/Erk2 (AF1891; Beyotime, 1:1000 for WB), and HRP-conjugated secondary antibody (SA00001-2, Proteintech, China, 1:5000 for WB, and 1:200 for IHC). A SuperEnhanced ECL kit (G3308, GBCBIO) was applied to amplify the exposure signal for WB analysis. The DAB horseradish peroxidase color development kit (G3433; GBCBIO) was employed for IHC analysis. The WB bands on the PVDF membrane were photographed using the gel imaging system (UVITEC, Cambridge, UK). The stained immunohistochemical pictures were recorded and analyzed using the OLYMPUS microscope (Tokyo, Japan). The grayscale analysis of WB bands and IHC pictures were performed using Image J software (version 1.52a, USA).

### Wound healing assays and transwell assay

The wound healing assays and transwell assay were performed as described previously 6. HCC cells (1 × 10^5^ cells/well) were plated in 6-wells plates and cultured to 70% confluence. After transfection, a linear wound was made for each well using a sterilized pipette tip and imaged with a microscope at 0 h and 48 h. A total of 200 μl HCC cells suspension (5 × 10^4^ cells) was added into the upper compartment of the transwell chamber (353097; FALCON, USA) with polycarbonate membrane coated with Matrigel (356234; Coring, USA) in advance. Then, 600 μl DMEM medium containing 20% FBS was added into the lower compartment. After incubation at 37 °C for 48 hours, the cells on the lower surface of the membrane were fixed, stained, imaged, and counted under a microscope (OLYMPUS, Tokyo, Japan).

### Animal experiments

In this study, all animals were treated and handled in accordance with the Animal Research: Reporting of *In vivo* Experiments (ARRIVE) guidelines and approved by The Medical Animal Care & Welfare Committee of Shantou University (SUMC2020-320). Twenty SPF grade male BALB/c-nu/nu mice (four weeks of age), purchased from the Guangdong Medical Laboratory Animal Center (Guangzhou, China), were raised under the environment of temperature (26°C-28°C), humidity (43%-47%), ventilation (12 times/h), and 12 h-12 h light/dark with free access to water and feed. For xenograft growth analysis, HCC cells (10^7^ cells/200 μL PBS) were injected subcutaneously into the armpits of mice (5 mice per group). The tumor's width and length were measured using vernier calipers once every two days, as well as the weight of mice. Then, the tumor volume was calculated based on the formula: tumor volume (mm^3^) = (width^2^ × length)/2. After 30 days, the mice were sacrificed by carbon dioxide suffocation. Then, the tumor growth curve was drawn, and the xenograft tumor tissues were harvested for WB and IHC analysis.

### Statistical analysis

All the data were expressed as mean ± standard deviation (SD). Triplicate independent experiments were performed for each assay. One-way ANOVA analysis was performed for statistical evaluation of the data. The paired t-test was performed for the comparison between HCC cancer tissues and matched para-carcinoma tissues. The independent Student's t-test was applied for the comparison of two independent samples. For all analyses, *P* < 0.05 was regarded as statistically significant. Unless otherwise noted, statistical diagrams were drawn using GraphPad Prism software (version 8.0.1, USA).

## Results

### Screening and construction of HCC ceRNA networks

**Figure 1:**
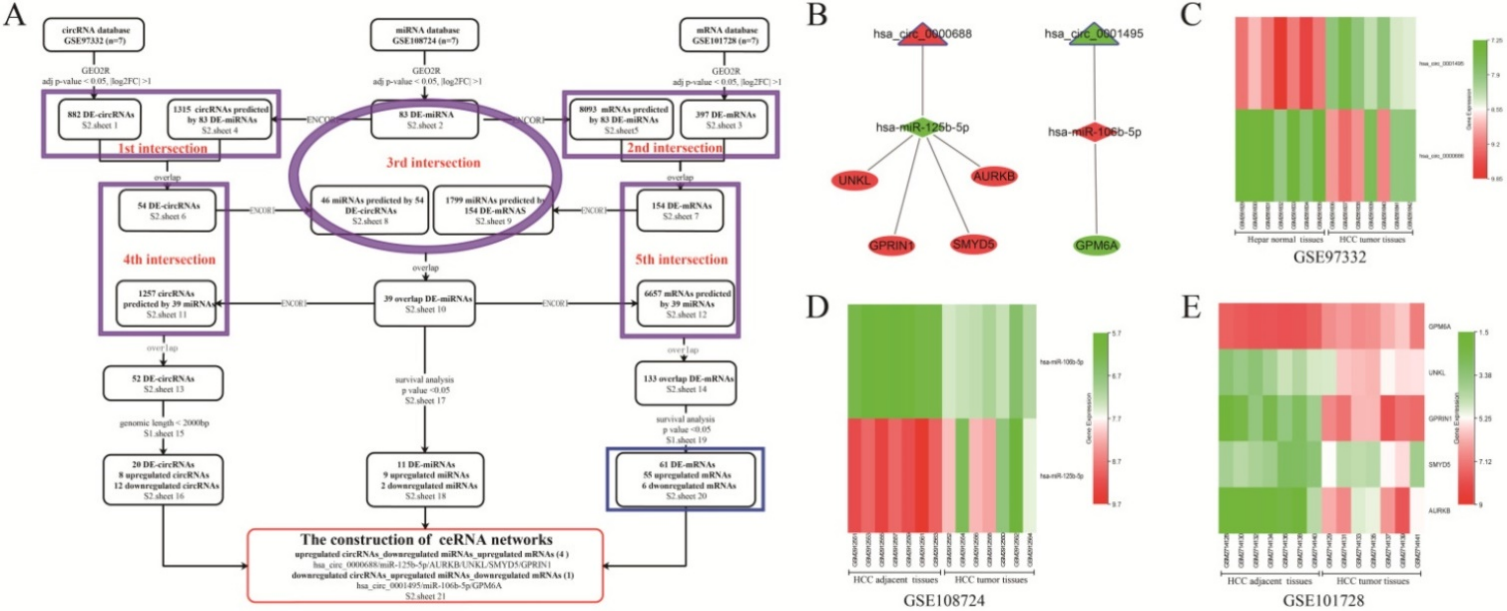
** The screening, construction, and composition of HCC related ceRNA network. (A)** The screening flowchart of the HCC related ceRNA network is composed of circRNA, miRNA, and mRNA. The purple ellipses and rectangles represent the intersection of the datasets covered by them, and the overlapping RNAs obtained were used for the following analysis. The blue rectangle represents the 61 overlapped mRNAs used for the GO and KEGG analysis. **(B)** The construction of 5 HCC related ceRNA networks. The triangles, diamonds, and ellipses represent circRNA, miRNA, and mRNA, respectively. The red and green colors represent up-regulation and down-regulation in HCC tissues, respectively. **(C)** The heatmaps of two circRNAs expressions in GSE97332. **(D)** The heatmaps of two miRNAs expression in GSE108724. **(E)** The heatmaps of five mRNAs expression in GSE101728 dataset.

The flowchart representing DE-RNAs screening and ceRNA networks construction is shown in Figure 1A. The traceable RNA lists were showed in Table S2 (sheets 1-21). As shown in the flowchart, a total of 882 DE-circRNAs, 83 DE-miRNAs, and 397 DE-mRNAs between HCC tissues and matched para-carcinoma tissues were screened. Moreover, 1315 circRNAs and 8093 mRNAs were predicted from 83 DE-miRNAs based on the ENCORI database. After the first and second intersection, 54 circRNAs and 154 DE-mRNAs were obtained and used to predict their target miRNAs. With the third intersection of miRNAs, 39 miRNAs overlapped, and prediction of their target circRNAs and mRNAs was made. Subsequently, the fourth (between 54 circRNAs and 1257 predicted circRNAs) and fifth (between 154 DE-mRNAs and 6657 predicted mRNAs) intersections were performed to acquire 52 DE-circRNAs and 133 DE-mRNAs, respectively. Furthermore, after removing circRNAs with genomic length more than 2000bp, and miRNAs and mRNAs not significantly correlated with HCC survival and prognosis, 20 DE-circRNAs (8 up-regulated and 12 down-regulated circRNAs), 11 DE-miRNAs (9 up-regulated and two down-regulated), and 61 DE-mRNAs (55 up-regulated and six down-regulated) were obtained for the construction of the ceRNA network. Finally, five visualized ceRNA regulatory networks were established based on two ceRNA network models: up-regulated circRNA/down-regulated miRNA/up-regulated mRNA (4 networks) or down-regulated circRNA/up-regulated miRNA/down-regulated mRNA (1 network) (Figure 1B). GO analysis of the 61 DE-mRNAs revealed that enriched biological process (BP) included cell division, cell proliferation, and regulation of cell cycle (Figure S1A). Changes in cellular component (CC) were mainly enriched in nucleus, cytosol, and nucleoplasm (Figure S1B), while molecular function (MF) mainly involved protein binding, protein C-terminus binding, and protein kinase activity (Figure S1C). KEGG pathway analysis showed that the 59 DE-mRNAs were mainly enriched in the MAPK signaling pathway, Oocyte meiosis, and cell cycle (Figure S1D). As shown in the heatmaps (Figures 1C-E), the hsa_circ_0001495, miR-125b-5p, and GPM6A were down-regulated in cancer tissues compared to their paired para-carcinomal tissues of HCC, whereas hsa_circ_0000688, miR-106b-5p, and four mRNAs (*UNKL, GPRIN1, SMYD5, AURKB*) were up-regulated. Finally, taking into account our team's time and energy limitations, the circCCNB1/miR-106b-5p/GPM6A ceRNA network was randomly selected for subsequent functional mechanism exploration and experimental verification. The results of UALCAN analysis showed that lower GPM6A expression was positively correlated with cancer stage, tumor grade, nodal metastasis status, and histological subtypes in HCC (Figures S1E-I). Importantly, underexpression of GPM6A correlated with poor survival of HCC patients (Figure S1J, *P*-value = 0.037). The online analysis results of ENCORI showed that the expression level of miR-106b-5p in HCC tissues was significantly higher than that of normal liver tissues (Figure S1K). Moreove, overexpression of miR-106b-5p also significantly correlated with poor survival of HCC patients (Figure S1L, *P*-value = 0.019). The result of co-expression analysis showed that miR-106b-5p expression was negatively correlated with GPM6A expression (Figure S1M, r = - 0.251 and *P*-value = 1.01E-06). Therefore, our team hypothesized that the circCCNB1/miR-106b-5p/*GPM6A* ceRNA network might play an important role in regulating the cell cycle and proliferation of HCC cells.

### Expression analysis of circCCNB1, miR-106b-5p, and GPM6A in HCC cells and clinical samples

**Figure 2:**
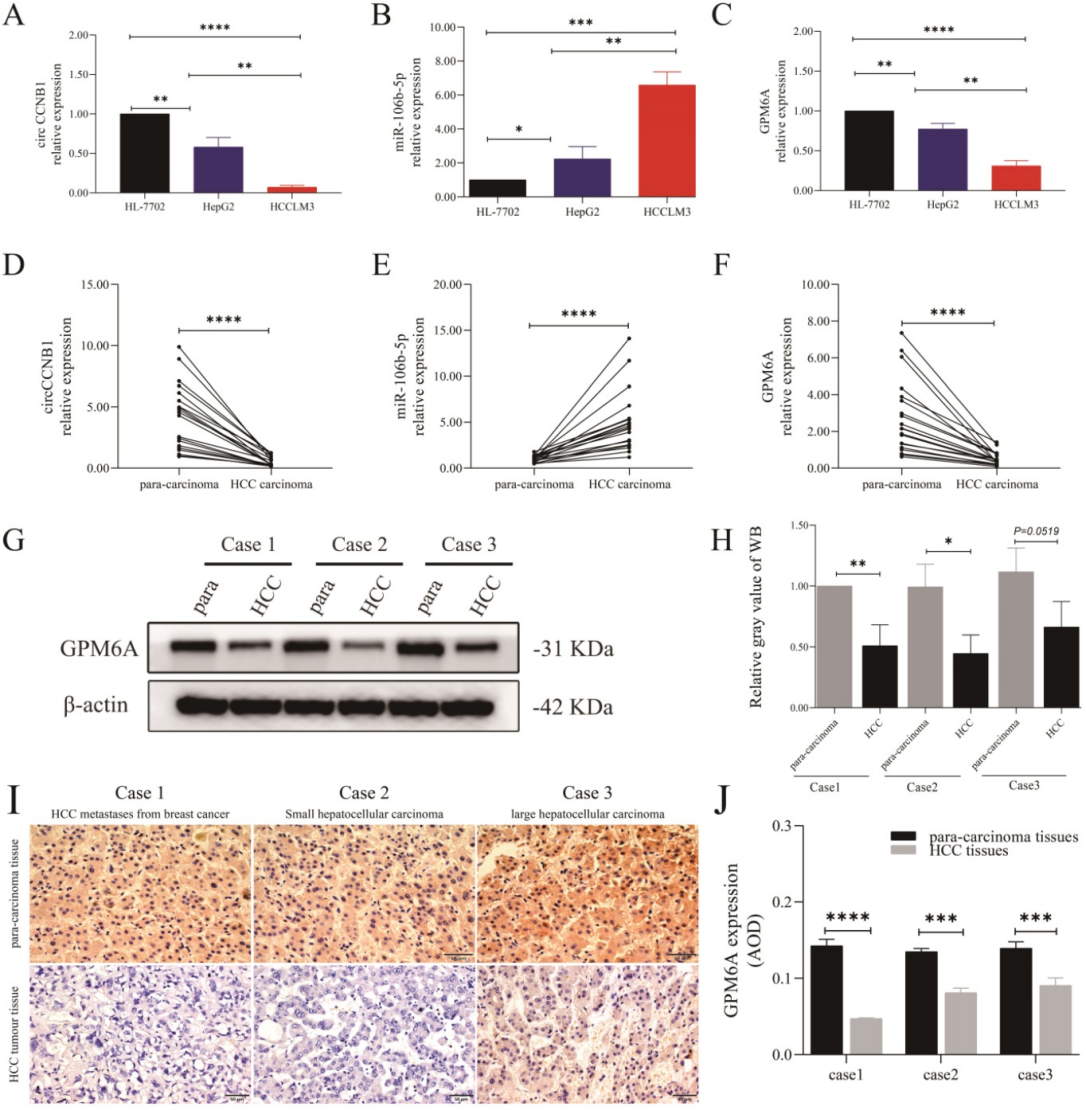
** The expression role of circCCNB1, miR-106b-5p, and GPM6A in the cells and tissues of HCC.** The expression level of circCCNB1 **(A)**, miR-106b-5p **(B)**, and GPM6A **(C)** in two HCC cell lines (HepG2 and HCCLM3) and one normal liver cell line (HL-7702) detected by qRT-PCR. The expression level of circCCNB1 **(D)**, miR-106b-5p **(E)**, and GPM6A **(F)** in twenty pairs of carcinoma and para-carcinoma tissues detected by qRT-PCR. **(G)**. The GPM6A expression levels of three pairs of HCC carcinoma and para-carcinoma tissues detected by WB. **(H)** Quantification from G. **(I)** The GPM6A expression levels in the carcinoma and para-carcinoma tissues of three pairs of HCC patients detected by IHC. **(J)** Quantification from I. One-way ANOVA analysis was used to compare the three RNAs expression between four cell lines. The two-independent t-test was used to compare the difference between any two cell lines. paired t-test was used to compare the difference between carcinoma and para-carcinoma tissues. *P<0.05, **P<0.01, ***P<0.001, ****P<0.0001.

The results of cell qRT-PCR showed that circCCNB1 and GPM6A were notably down-regulated in HepG2 and HCCLM3 cells compared to HL-7702 cells, while miR-106b-5p was up-regulated (Figures 2A-C). Notably, the expression level of circCCNB1/GPM6A was the lowest, and the expression of miR-106b-5p was the highest in HCCLM3 cells compared to HepG2 cells. qRT-PCR demonstrated circCCNB1/GPM6A underexpression and miR-106b-5p overexpression in cancer tissues compared to the para-carcinoma tissues in twenty HCC patients (Figures 2D-F). Next, three pairs of cancer and para-carcinoma tissues of three different pathological types, including metastatic liver tumor from breast cancer, small HCC, and large HCC, were utilized for WB and IHC analysis. WB analysis (Figures 2G and H) and IHC analysis (Figures 2I and J) showed that the GPM6A expression was much lower in cancer tissues than para-carcinoma tissues in HCC. Moreover, GPM6A was mainly localized in the cytoplasm of HCC cells by visual assessment.

### The effects of deregulated miR-106b-5p on GPM6A expression, proliferation, apoptosis, migration, and invasion of HCC cells

**Figure 3:**
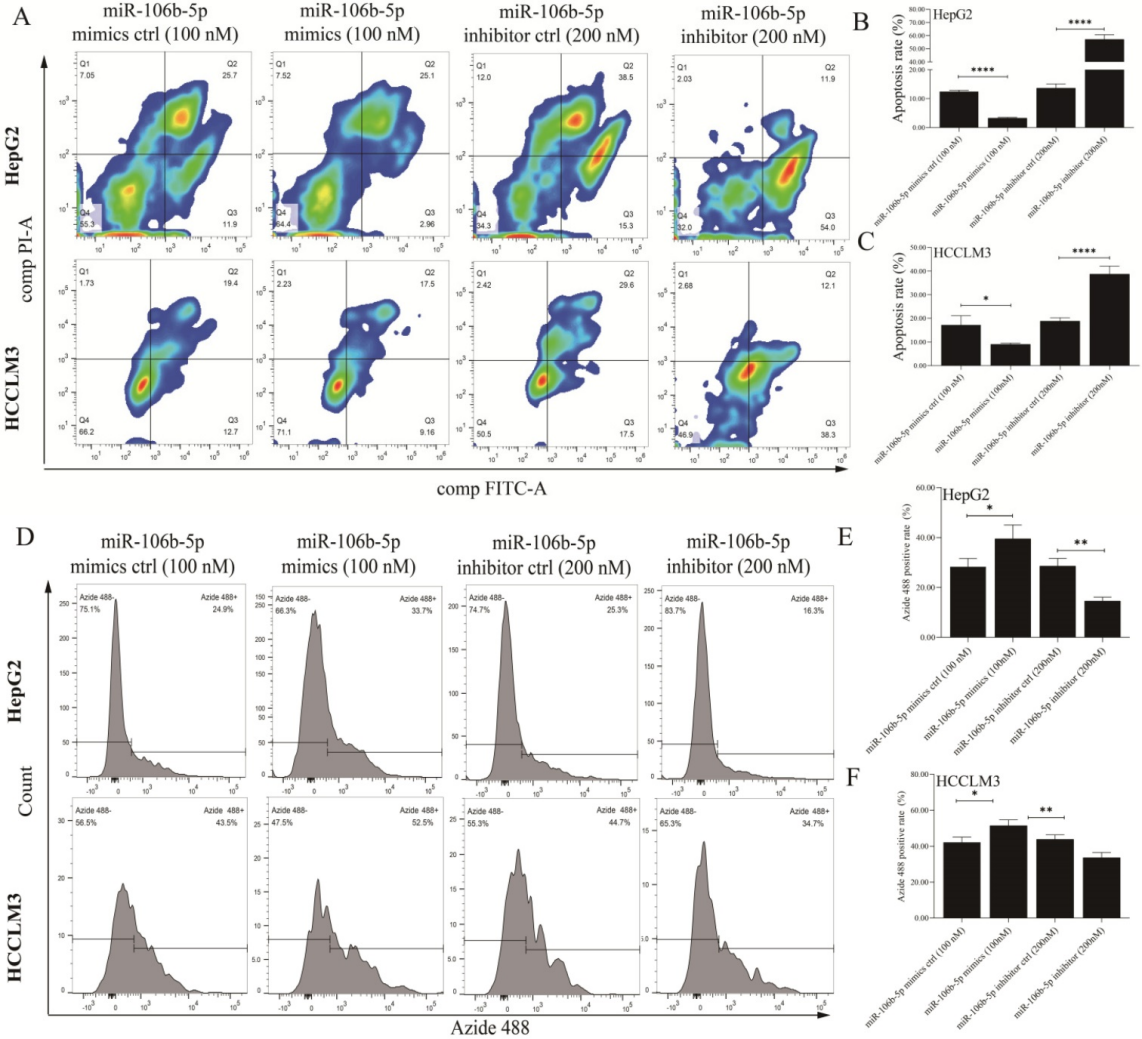
** The upregulated expression of miR-106b-5p notably inhibited apoptosis and promoted proliferation in HCC cells.** After the transfection of miR-106b-5p mimics and inhibitors, The cell apoptosis rate **(A)** and proliferation **(D)** of HCC cells were measured using the assays of FITC/PI and EdU methods, respectively. **(B, C)** Quantification from A. **(E, F)** Quantification from D. The two independent t-tests were used to compare the difference between any two groups. *P<0.05, **P<0.01, ****P<0.0001.

The miR-106b-5p mimics, miR-106b-5p inhibitor, and their negative control were synthesized to explore the effects of deregulated miR-106b-5p on GPM6A expression and HCC progression. After HepG2 and HCCLM3 cells were transfected with mimics (50 nM and 100 nM) and inhibitors (100nM and 200 nM) continued to be cultured for 48 h, qPCR and WB were performed to detect the expression level of miR-106b-5p and GPM6A. The miR-106b-5p qPCR results of HCC cells transfected with miRNA mimics showed that the miR-106b-5p expression was increased with a positive correlation with the concentration of mimics (Figure S2A). The qPCR results of HCC cells transfected with miR-106b-5p productions demonstrated that the GPM6A expression was negatively and positively correlated with the concentration of mimics and inhibitors (Figure S2B). The WB results of HCC cells transfected with miR106b-5p mimics and inhibitors showed that the expression of GPM6A was negatively correlated with the concentration of mimics and positively correlated with the concentration of inhibitors (Figures S2C-E). After the above experimental validation, 100 nM and 200nM were selected as the final concentration of mimics and inhibitors of miR-106b-5p for subsequent transfections, respectively. Firstly, the analysis of Annexin V-FITC/PI apoptosis was performed to explore the effect of miR-106b-5p productions on the apoptosis of HCC cells. The results revealed that the overexpressed and underexpressed miR-106b-5p significantly inhibited and enhanced the apoptosis process of HCC cells (Figures 3A-C). Moreover, the EdU test was applied to evaluate the role of miR-106b-5p in HCC cell proliferation (Figures 3D-F). The EdU results indicated that the higher miR-106b-5p expression strongly promoted the proliferation of HCC cells, while lower miR-106b-5p expression decreased the proliferation of HCC. Furthermore, the wound healing assay was implemented to assess the effect of the abnormal miR-106b-5p expression on the migration of HCC cells. The results showed that the up-regulated and down-regulated miRNA significantly accelerated and weakened the ability of HCC cell migration (Figures S3A-C). Finally, the transwell assay was performed to explore the influence of aberrant miR-106b-5p expression on HCC cell invasion. The activation of miR-106b-5p expression, caused by mimics transfection, actively promoted the invasion of HCC cells, which was the opposite of inactivation of miR-106b-5p expression caused by inhibitors (Figures S3D-F).

### The effects of deregulated circCCNB1 on miR-106b-5p/GPM6A expression, cell cycles, and Clone formation ability of HCC cells

**Figure 4:**
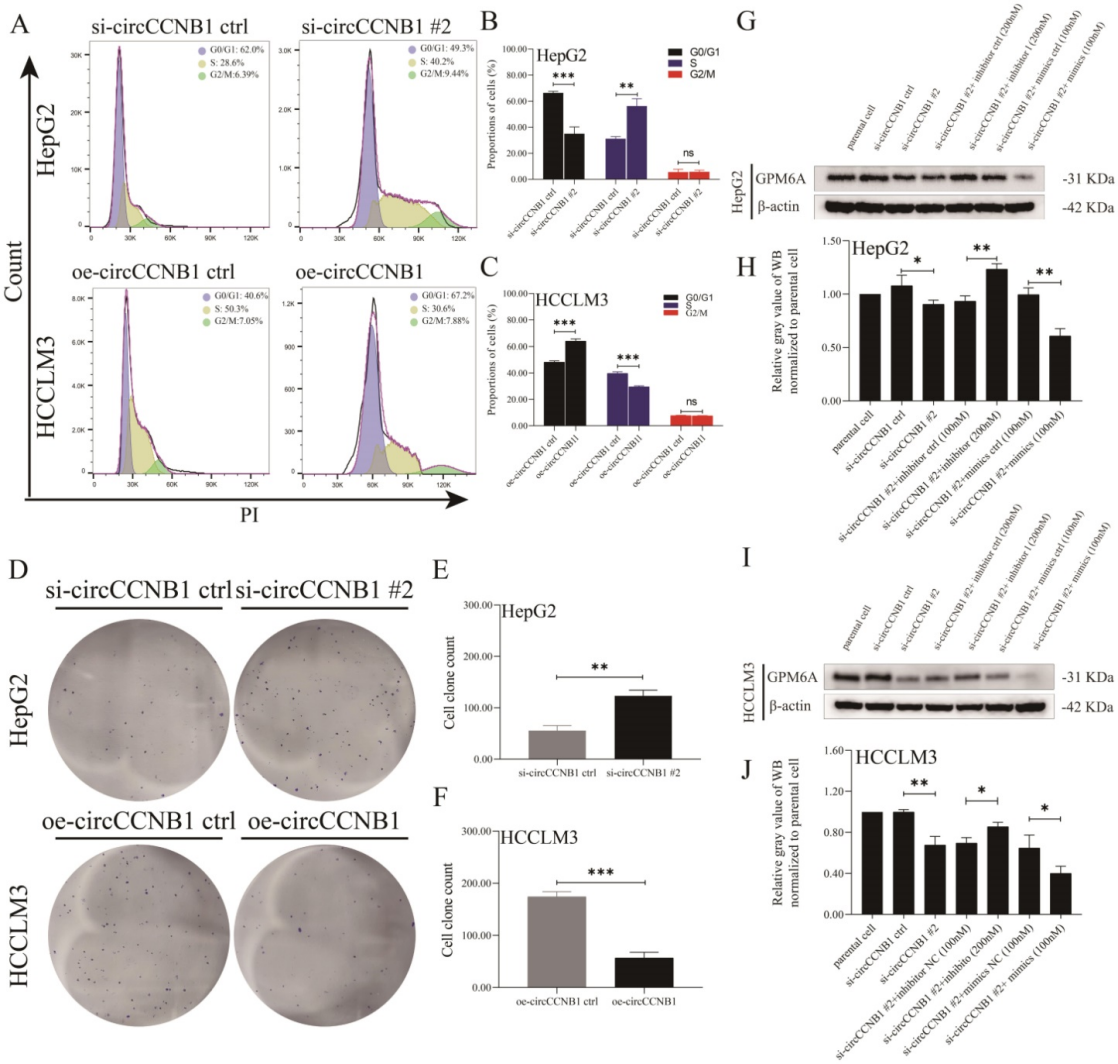
** Aberrant expression of circCCNB1 significantly regulated cell cycle and clonality of HepG2 and HCCLM3 cells. (A)** The G1-S transition of the cell cycle was, respectively, promoted and blocked in HepG2 (circCCNB1 silent expression) and HCCLM3 (circCCNB1 overexpression) cells determined by PI analysis. **(B, C)** Quantification from A. **(D)** The underexpression and overexpression of circCCNB1 significantly enhanced and inhibited the clonal formation ability of HepG2 and HCCLM3 cells. **(E, F)** Quantification from D. **(G-J)** The efficiency of circCCNB1 silencing and miR-106-5p intervention in hepG2 and HCCLM3 cells was determined by GPM6A WB analysis. After silencing of circCCNB1, miR-106b-5p inhibitor or mimics was added to hepG2 **(G)** and HCCLM3 **(I)** cell culture. The GPM6A protein expression level was analyzed by Western blot. **(H)** Quantification from G. **(J)** Quantification from I. Data was derived from the results of three independent repeated experiments (Mean ± SD). The two independent t-tests were utilized to compare cell cycle and clonal formation ability between any two groups. ns represents not significant, *P<0.05, **P<0.01, ***P<0.001, ****P<0.0001.

To explore the role of circCCNB1 on miR-106b-5p/GPM6A expression, the lentivirus vectors of circCCNB1 silencing (si-circCCNB1 #1, #2, and #3) and overexpression (oe-circCCNB1) were designed and constructed by Genechem company. After transfection with si-circCCNB1 and oe-circCCNB1 into HepG2 and HCCLM3 cells, the silencing and overexpression efficiency of si-circCCNB1 and oe-circCCNB1 were verified using qRT-PCR. The results of circCCNB1 qRT-PCR revealed that compared to the si-circCCNB1 negative control (si-circCCNB1 ctrl), all the transfected si-circCCNB1 down-regulated the expression of circCCNB1 in HepG2 and HCCLM3 cells. However, compared with si-circCCNB1 #1 and #3, si-circCCNB1 #2 significantly inhibited the expression of circCCNB1 (Figure S4A). miR-106b-5p expression in HCC cell lines was determined using qRT-PCR to verify the miRNA sponge function of circCCNB1. The results of miR-106b-5p qRT-PCR showed that circCCNB1 silencing and overexpression did not affect the expression levels of miR-106b-5p (Figure S4B). Moreover, to confirm the effects of circCCNB1/miR-106b-5p/GPM6A, mRNA and protein expression levels of GPM6A were evaluated using qRT-PCR and WB. The mRNA and protein expression levels of GPM6A were notably down-regulated and up-regulated by si-circCCNB1 #2 and oe-circCCNB1 transfection (Figures S4C-F). Thus, it was suggested that circCCNB1 might protect GPM6A from downregulation by miR-106b-5p in HCC cells. Considering the excellent silencing results of circCCNB1, si-circCCNB1 #2 was selected for subsequent function experiments. Furthermore, to investigate the effect of circCCNB11 on HCC progression, cell cycles and cell clone formation assays were performed for HepG2 and HCCLM3 cells transfected with si-circCCNB1 #2, oe-circCCNB1, and their negative controls. As shown in Figures 4A-C, down-regulated circCCNB1 in HepG2 cells caused by si-circCCNB1 #2 transfection markedly promoted G1-S transition and increased the proportion of S-phase cells, where up-regulated circCCNB1 in oe-circCCNB1 transfected HCCLM3 cells blocked the G1-S transition and decreased the proportion of the S-phase cells. Similarly, circCCNB1 silencing significantly improved the cell clone formation ability of HepG2 cells, while circCCNB1 overexpression notably weakened the cell clone formation ability of HCCLM3 cells (Figures 4D-F). In addition, miR-106b-5p inhibitor or mimics were utilized to assess whether the downregulation or upregulation of miR-106b-5p could block and aggravate the under-expression of GPM6A caused by circCCNB1 silencing. Interestingly, miR-106b-5p inhibitors significantly obstructed the circCCNB1 silencing-mediate downregulation of GPM6A in HepG2 and HCCLM3 cells (Figures 4G-J). Therefore, we concluded that circCCNB1 inhibited HCC progression as the sponge of miR-106-5p to remove the miR-106b-5p carcinogenesis via circCCNB1/miR-106b-5p/GPM6A ceRNA network.

### Targeted binding of miR-106b-5p VS GPM6A and miR-106b-5p VS circCCNB1

**Figure 5:**
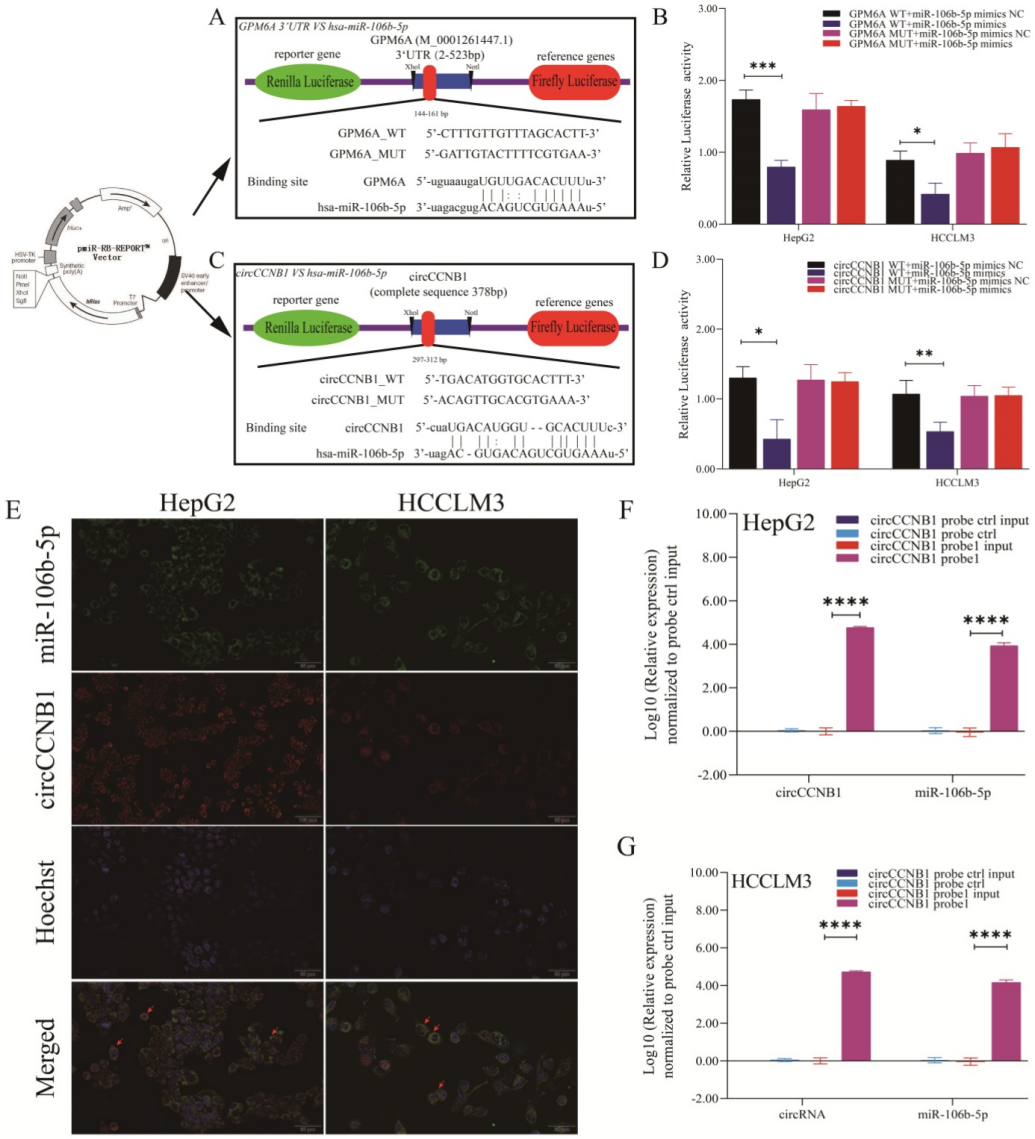
** Targeted binding of circCCNB1 VS miR-10106b-5p and miR-106b-5p VS GPM6A. (A, C)** Schematic diagram of report vector construction of dual-luciferase reporter assay. **(A)** The constructed vector for the dual-luciferase reporter of GPM6A 3' UTR VS miR-106b-5p. **(C)** The constructed vector for the dual-luciferase reporter of circCCNB1 vs. miR-106b-5p. **(B)** Quantification from the right-upper of A. **(D)** Quantification from the right-lower of C. **(E)** circCCNB1 co-localized with miR-106b-5p in HepG2 and HCCLM3 cells detected by FISH. miR-106b-5p was pulled down and enriched with a circCCNB1 specific probe in HepG2 **(F)** and HCCLM3 **(G)** cells then detected by qPCR. Data were derived from the results of three independent repeated experiments (Mean ± SD). The bar of the pictures (E) represents 50 μm. *P<0.05, **P<0.01, ***P<0.001, ****P<0.0001.

The dual-luciferase report experiment was designed and performed to explore whether miR-106b-5p suppressed GPM6A expression by binding to the GPM6A 3'UTR region and the target sponge binding between circCCNB1 and miR-106b-5p. The inserted sequences of GPM6A 3'UTR WT and MUT vectors and the binding site of miR-106b-5p on GPM6A 3'UTR predicted by MiRanda algorithm were shown in Figure 5A. The inserted sequences of circCCNB1 WT and MUT vectors and the binding site of miR-106b-5p on circCCNB1 predicted by circBase algorithm were shown in Figure 5C. After the transfection of the vector and mimics, the luciferase intensity was notably reduced when hepG2 and HCCLM3 cells were co-transfected with the WT vector of GPM6A 3'UTR and miR-106b-5p mimics, while no changes were observed after co-transfection with the MUT vector of GPM63A and miR-106b-5p mimics (Figure 5B). In addition, the result of circRNA/miRNA luciferase reporter assays showed that the luciferase intensity was also significantly decreased when HepG2 and HCCLM3 cells were co-transfected with circCCNB1 WT vector and miR-106b-5p mimics (Figure 5D). The co-location of circCCNB1 and miR-106b-5p was explored using the FISH assay. Figure 5E confirmed that the circCCNB1 was co-localized with miR-106b-5p in the cytoplasm of HepG2 and HCCLM3 cells. To validate the target binding of circCCNB1/miR-106b-5p, the circRIP assay was also performed using a biotin-labeled circCCNB1 specific probe. The miRNAs were extracted from the complex composed of streptavidin-coated magnetic beads-biotin labeled circCCNB1 probe-circCCNB1-binding RNAs and determined by qRT-PCR. circCCNB1 and miR-106b-5p expression were determined from the circRNA pull-down complex, not from the supernatant in HepG2 and HCCLM3 cells (Figures 5F and 5G). Thus, it was confirmed that the direct target binding between miR-106b-5p vs. circCCNB1 and miR-106b-5p vs. GPM6A existed in HCC cells lines.

### CircCCNB1 silencing promoted the tumor growth *in vivo*

**Figure 6:**
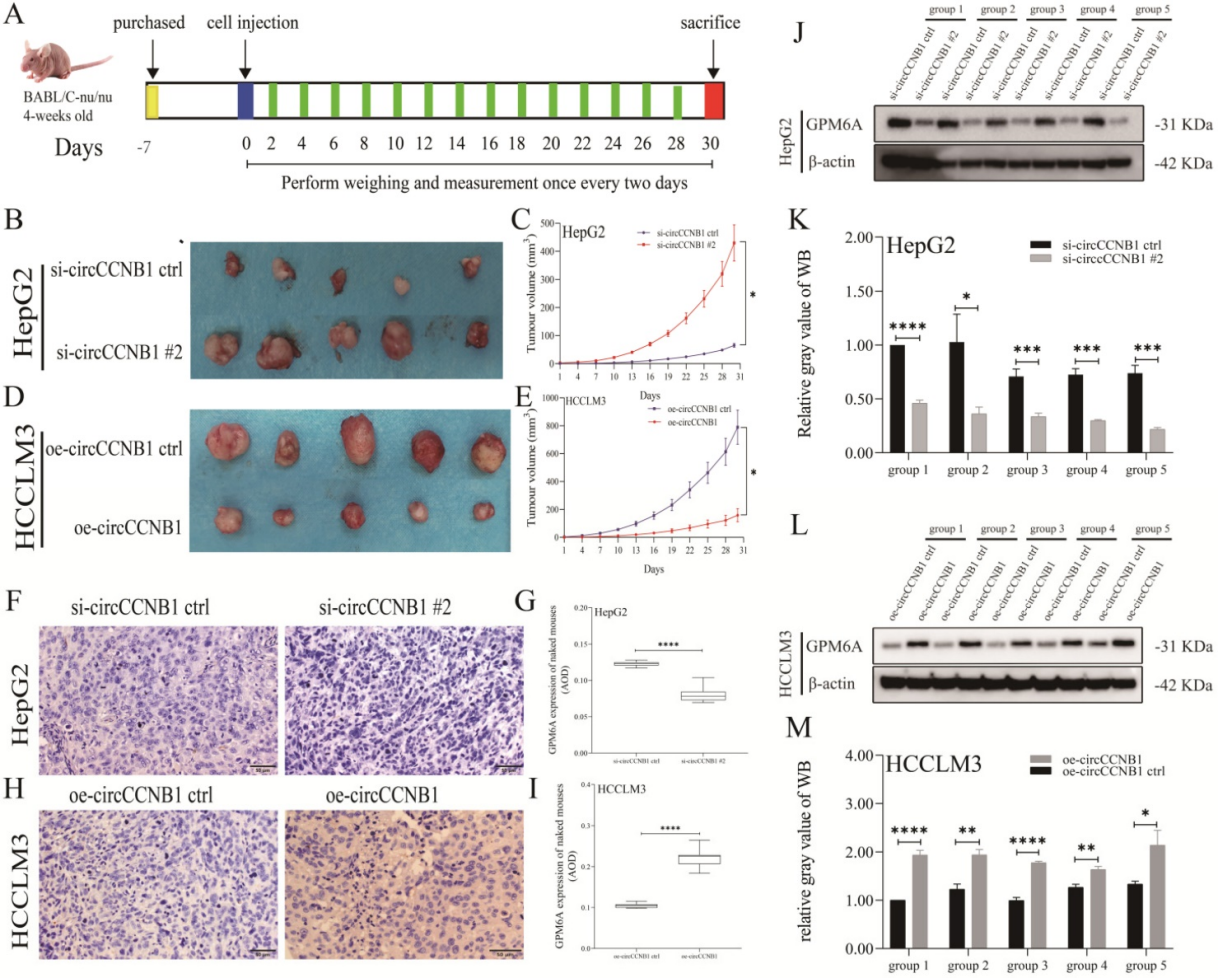
** CircCCNB1 down expression promotes tumor growth *in vivo*. (A)** Schema of *in vivo* xenograft models. **(B, C)** The xenograft tumors of circCCNB1 silencing HepG2 grew faster and bigger than that of circCCNB1 silencing ctrl. **(D, E)** The xenograft tumors of circCCNB1 overexpressing HCCLM3 cells grew slower and smaller than that of circCCNB1 overexpressing ctrl. The two-way ANOVA was applied to analyze the tumor growth difference between the two groups. **(F, H)** IHC staining analysis of GPM6A expression for the resected xenograft tumor tissues of five paired mice. **(G, I)** Quantification from F and H, respectively. The two-independent t-test analyzed the difference of IHC pictures' AOD value. **(J, L)** WB analysis of GPM6A expression for the resected xenograft tumor tissues of five paired mice. **(K, M)** Quantification from J and L. Data of the three independent experiments was represented as Mean ± SD. *P<0.05, ****P<0.0001.

To explore the effects of circCCNB1 on tumor growth *in vivo*, the circCCNB1 silencing HepG2 cells and circCCNB1 overexpressing HCCLM3 cells were subcutaneously injected into the armpit of nude mice (Figure 6A). In the following 30 days, the xenograft size was measured to generate tumor growth curves. Figures 6B and 6C showed that circCCNB1 silencing resulted in faster tumor volume growth in the HepG2 xenograft mouse model, while circCCNB1 overexpression had the opposite effects in HCCLM3 xenograft mouse models (Figures 6D and 6E). Moreover, the tumor tissues from the xenograft mouse model were used to perform GPM6A IHC and WB assays. GPM6A proteins were stained into brown-yellow particles in IHC pictures and mainly located in the cytoplasm of HCC cells (Figures 6F and 6H). The IHC (Figures 6G and 6I) and WB (Figures 6J-6M) results revealed that GPM6A protein level was significantly higher in mice of si-circCCNB1 ctrl and oe-circCCNB1 xenograft models, compared with those of si-circCCNB1 #2 and oe-circCCNB1 ctrl xenograft models. These results confirmed that circCCNB1 silencing could promote xenograft growth *in vivo* through downregulation of GPM6A expression.

### Downregulation of GPM6A mediated by circCCNB1 silencing activated AKT/ERK signaling pathway through up-regulating DYNC1I1

**Figure 7:**
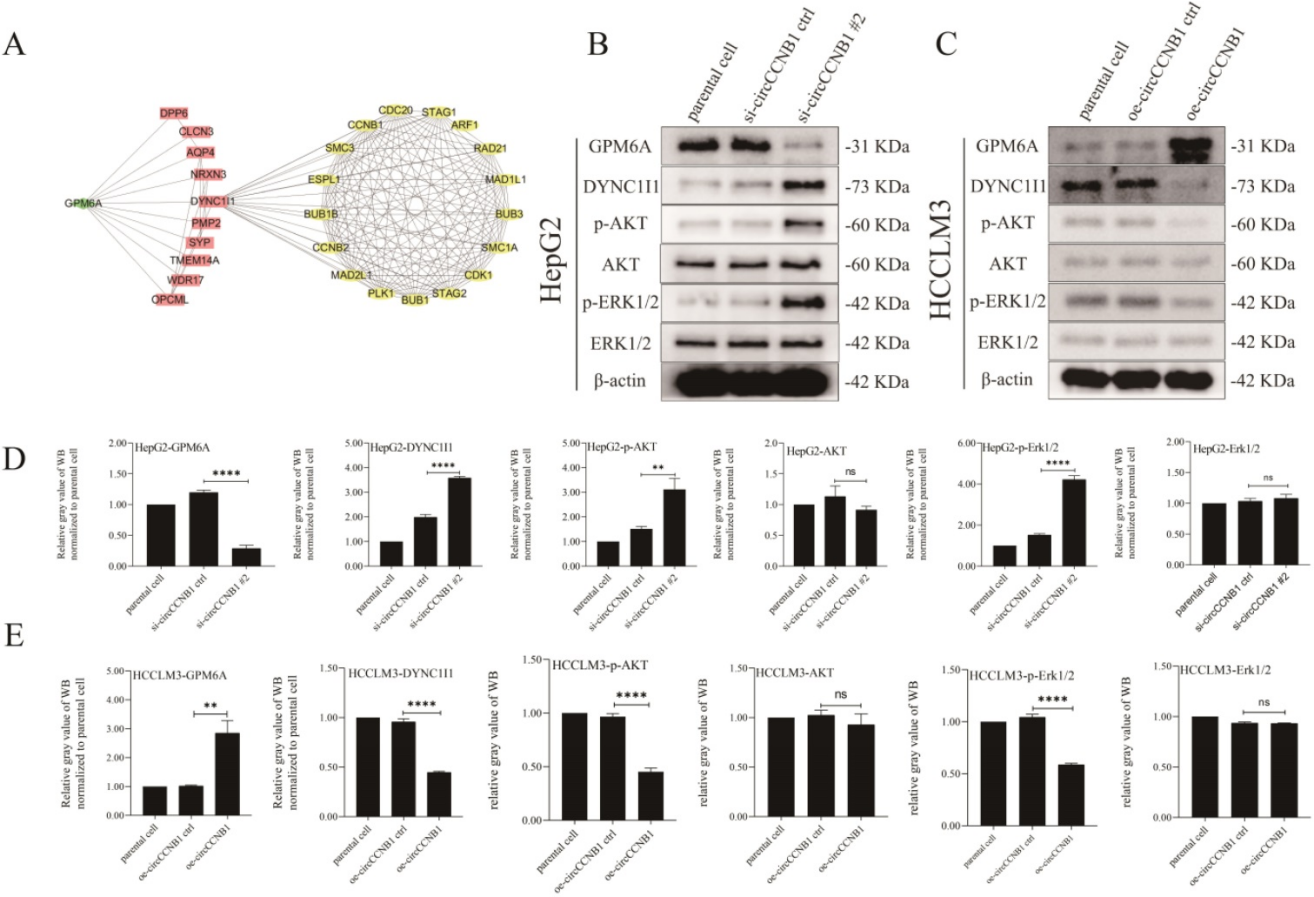
** Silent expression of circCCNB1 promotes the cell cycle of HCC cells by down-expression of GPM6A and up-expression of DYNC1I1 and activation of AKT and ERK signaling pathway. (A)** The PPI networks. The PPI network was constructed by GPM6A (green rhombus), ten genes interacting with GPM6A predicted by STRING (pink rectangles), and 145 cell cycle-related genes (yellow Ellipse) derived from PathCards using Cytoscape software. **(B)** CircCCNB1 silencing inhibited GPM6A expression and up-regulated DYNC1I1 expression by activating the AKT/ERK signaling pathway in HepG2 cells. **(C)** CircCCNB1 overexpressing promoted GPM6A expression and reduced DYNC1I1 expression by inhibiting the activation of AKT/ERK signaling pathway in HCCLM3 cells. **(D)** Quantification from B. **(E)** Quantification from C. Data of the three independent experiments was represented as Mean ± SD. The two-independent t-test was applied for comparing the difference of the gray value of the WB band between two groups (D, E). ns represents not significant, **P<0.01, ****P<0.0001.

The results of previous KEGG pathway analysis showed that the mRNAs involved in the construction of the ceRNA network were mainly enriched in the MAPK signaling pathway, which was one of the most common pathways of cell cycle regulation. To explore the effects of GPM6A on the cell cycle, ten genes interacting with GPM6A were utilized to establish PPI networks with GPM6A and 145 cell cycle-related genes derived from PathCards (Figure 7A). Surprisingly, the PPI networks showed that *GPM6A* might regulate seventy cell cycle-related genes through DYNC1I1. Thence, DYNC1I1, as the only link gene between GPM6A and cell cycle-related genes, spontaneously attracted our great attention. The results of UALCAN analysis showed that* DYNC1I1* overexpression was positively correlated with cancer stage, tumor grade, nodal metastasis status, and histological subtypes of HCC (Figures S5A-E). In addition, the co-expression analysis stated that GPM6A expression was negatively correlated with DYNC1I1 in HCC patients (Figure S5F). However, the K-M survival analysis showed that the *DYNC1I1* overexpression did not correlate with the poor survival of HCC patients (Figure S5G). To further explore the role of circCCNB1 on the MAPK signaling pathway, the expression of phosphorylated and unphosphorylated AKT and ERK1/2 were determined using circCCNB1 silencing HepG2 cells and circCCNB1 overexpressing HCCLM3 cells by WB assay. Figures 7B and 7D showed that the expression levels of *DYNC1I1, p-AKT,* and *p-ERK1/2* significantly increased in circCCNB1 silencing HepG2 cells compared with si-circCCNB1 ctrl. No change in the expression of *AKT* and *ERK1/2* was observed. Figures 7C and 7E showed that the levels of DYNC1I1, p-AKT, and p-ERK1/2 notably reduced in circCCNB1 overexpressing HCCLM3 cells with no change in the expression of AKT and ERK1/2. Therefore, it was concluded that GPM6A inhibition induced by circCCNB1 silencing could activate the AKT/ ERK signaling pathway to participate in the cell cycle regulation of HCC cells by enhancing the DYNC1I1 expression.

## Discussion

Studies on the effect of circRNA/miRNA/mRNA ceRNA networks on carcinogenesis and cancer progression are in full swing 18. Since the function and mechanism of ceRNA networks in HCC development are still unknown, three HCC related GEO datasets were utilized to screen the DE-circRNAs, DE-miRNAs, and DEmRNAs between paired HCC and matched para-carcinoma tissues. After a series of target RNA prediction, intersection, and filtering, the ceRNA networks of hsa_circ_0000688/miR-125b-5p/UNKL/GPRIN1/SMYD5/AURKB and hsa_circ_0001495/miR-106b-5p/GPM6A were constructed using Cytoscape software. Unfortunately, the circCCNB1/miR-106b-5p/GPM6A ceRNA network was randomly selected for subsequent experimental validation and mechanism exploration *in vitro and in vivo*. It has been reported that circCCNB1, which exerts anti-aging effects in cellular senescence 19, could inhibit the exacerbation of breast cancer induced by p53 mutation 20. Current studies have found that miR-106b-5p upregulation could promote the proliferation and metastasis of HCC cells by the negative regulation of its target genes PTEN and RUNX3 21, 22. Moreover, the ceRNA networks, constructed by miR-106b-5p and its upstream/downstream circRNAs/mRNAs, played an essential role in the pathogenesis and prognosis of epilepsy 23, renal cell carcinoma 24, and colon cancer 25. In addition, GPM6A has also been used as a cell marker to distinguish hepatic stellate cells and mesenchymal cells and was significantly correlated with liver fibrosis. To the best of our knowledge, the role of circCCNB1/miR-106b-5p/GPM6A ceRNA network in HCC has not been previously reported. This study was more innovative since we explored, constructed, and verified other HCC related ceRNA networks.

In the present study, it was found that circCCNB1 and GPM6A were significantly down-regulated in HCC cells or HCC tissues, while the expression of miR-106b-5p was the opposite, which was consistent with the results of our GEO2R analysis. In addition, it was confirmed that miR-106b-5p overexpression could significantly promote the proliferation, metastasis, and invasion of HCC cells by weakening GPM6A expression. In the meantime, circCCNB1 silencing could significantly inhibit the GPM6A expression and increase the proportion of the S-phase cells, the cloning formation ability, and xenograft growth of HCC cells, with no effect on the expression of miR-106b-5p. Subsequently, the results of the dual-luciferase assay confirmed that miR-106b-5p could directly bind to the 3'UTR region of GPM6A to down-regulate the GPM6A expression. Also, the miR-106b-5p, co-localizing with circCCNB1 in the cytoplasm, could be directly pulled down by the circCCNB1 probe confirmed by circRIP assay. Presumably, the miR-106b-5p and circCCNB1 may play a vital role in the post-translational regulation of GPM6A. Hence, it was concluded that circCCNB1 acting as miR-106b-5p sponge could protect GPM6A from the degradation induced by direct binding of miR-106b-5p to 3'UTR of GPM6A.

Previous reports had confirmed that stably expressed miRNAs in plasma had great potential as biomarkers for HCC diagnosis and treatment 26. Furthermore, circular RNAs, enriched in circulating exosomes of HCC patients, also play an essential role in the HCC diagnostic and therapeutic 27. A small group of exonic circRNAs (ecircRNAs) serves as miRNA sponges to competitively regulate the expression of miRNA-targeted mRNA 5. Later on, the ceRNA networks composed of circRNAs/miRNAs/mRNAs were found to perform crucial roles in the occurrence and development of HCC 18. In the present study, the HCC-related circRNA/miRNA/mRNA networks were constructed based on the hypothesis of crosstalks among ceRNA, mRNA, and circRNA via miRNA response elements (MRE). Furthermore, Chen L et al. used a similar ceRNA strategy for constructing the hsa_circ_0077210/hsa-miR-92b-3p/ACADL/CPEB3 ceRNA networks 18. Most regrettably, some necessary validation experiments were not designed to confirm the regulatory mechanism of the above-mentioned ceRNA networks in the development and progression of HCC. The screening criteria for length of spliced circRNA was set as less than 2000bp to remove the longer circRNAs, which were easy to break off and difficult to perform functional experiments. Convincingly, the experiments of cirRIP, dual-luciferase reporter experiment, and FISH were designed to explore and provide sufficient evidence of targeted binding or interaction between circCCNB1, miR-106b-5p, and GPM6A.

In our study, another significant finding was that GPM6A underexpression, induced by circCCNB1 silencing, activated the AKT/ERK signaling pathway to participate in the cell cycle regulation of HCC cells by up-regulating DYNC1I1 expression. Previous studies confirmed that the lack of DYNC1I1 could lead to the occurrence of neuronal autophagy, which was highly correlated to diseases such as aging and neuronal atrophy 28. In addition, some studies have reported that the upregulation of DYNC1I1 could inhibit the apoptosis of cancer cells and promote the proliferation and migration of cancer cells 29. DYNC1I1, the binding subunit of cytoplasmic dynein, could assist the cytoplasmic dynein-mediated transport of P65 to the nucleus, following which P65 could promote the expression of IL-6 and activate the STAT3 phosphorylation to promote the proliferation and metastasis of gastric cancer cells 30. Moreover, DYNC1I1 upregulation could also enhance its downstream TNPO2 expression through SP1 overexpression to promote the proliferation and invasion of gastric cancer cells 29. This study remarkably found that circCCNB1 silencing induced GPM6A under-expression and DYNC1I1 overexpression to promote the proliferation and invasion of HCC cells by activating the AKT/ERK signaling pathways. Although it was found that the protein interaction and negative regulatory relationship between GPM6A and DYNC1I1 existed based on the results of PPI and co-expression analysis, the design and implementation of more targeted experiments are also needed to verify the interactions between circCCNB1/GPM6A/other undiscovered RNAs and DYNC1I1, which will be the focus of our future studies.

In summary, our results showed that the expression of circCCNB1 and GPM6A was significantly down-regulated in HCC cells and cancer tissues compared to the normal liver cells and para-carcinoma tissues, while the expression of miR-106b-5p was notably up-regulated. Functionally and mechanistically, circCCNB1 silencing indirectly down-regulated the expression of GPM6A by weakening the sponge adsorption of miR-106b-5p to promote the growth of HCC. Subsequently, GPM6A underexpression promoted the expression of its downstream gene DYNC1I1 and activated the AKT/ERK signaling pathways to modulate cell cycles in HCC. Therefore, our data demonstrated that the circCCNB1/miR106b-5p/GPM6A ceRNA network played a critical role in the occurrence and development of HCC, which may provide a new strategy for future HCC clinical treatment.

## Supplementary Material

Supplementary figures and table 1.

Supplementary table 2.

## Abbreviations

CircRNAs: circular RNAs
HCC: hepatocellular carcinoma
ceRNA: competitive endogenous RNA
DE: differential expression
MRE: miRNA recognition element
FISH: fluorescence *in situ* hybridization
circRIP: circRNAS *in vivo* precipitation
WB: western blotting
IHC: Immunohistochemistry
