# CircCCNB1 silencing acting as a miR-106b-5p sponge inhibited GPM6A expression to promote HCC progression by enhancing DYNC1I1 expression and activating the AKT/ERK signaling pathway: Erratum

**DOI:** 10.7150/ijbs.72651

**Published:** 2022-03-22

**Authors:** Yan-ming Liu, Yue Cao, Ping-sen Zhao, Liang-yin Wu, Ya-min Lu, Yu-long Wang, Jia-feng Zhao, Xin-guang Liu

**Affiliations:** 1 Guangdong Provincial Key Laboratory of Medical Molecular Diagnostics, Institute of Aging Research, Guangdong Medical University, Dongguan, Guangdong, China.; 2 Department of Clinical Laboratory, YueBei People's Hospital, Shaoguan, Guangdong, China.; 3 The Third Clinical Medical College, Guangzhou University of Chinese Medicine, Guangzhou, Guangdong, China.; 4 Department of Medical Technology, Medical College of Shaoguan University, Shaogguan, Guangdong, China.; 5 Department of Anesthesiology, YueBei People's Hospital, Shaoguan, Guangdong, China.; 6 Department of Hepatobiliary Surgery, YueBei People's Hospital, Shaoguan, Guangdong, China.

## Correction 1

In Figure 1A, the number of *S1.sheet 15* and *S1.sheet 19* was wrong, the correct number was ***S2.sheet 15*** and ***S2.sheet 19***, respectively. The correct picture is displayed below.

## Correction 2

In Figure 3F, the sign (*) of significant difference is not marked in the proper position, the correct picture is displayed below.

## Correction 3

In Figure 5E, the ***circCCNB1's picture of HepG2*** cell, which did not match the other three pictures (miR-106b-5p, Hoechst, and merged), was misused. The correct picture is displayed below.

## Correction 4

In Figure S4E of the Supplementary figures and tables file (S1), the signs (*) of significant difference are not marked in the proper position. The correct picture is displayed below.

## Figures and Tables

**Figure A:**
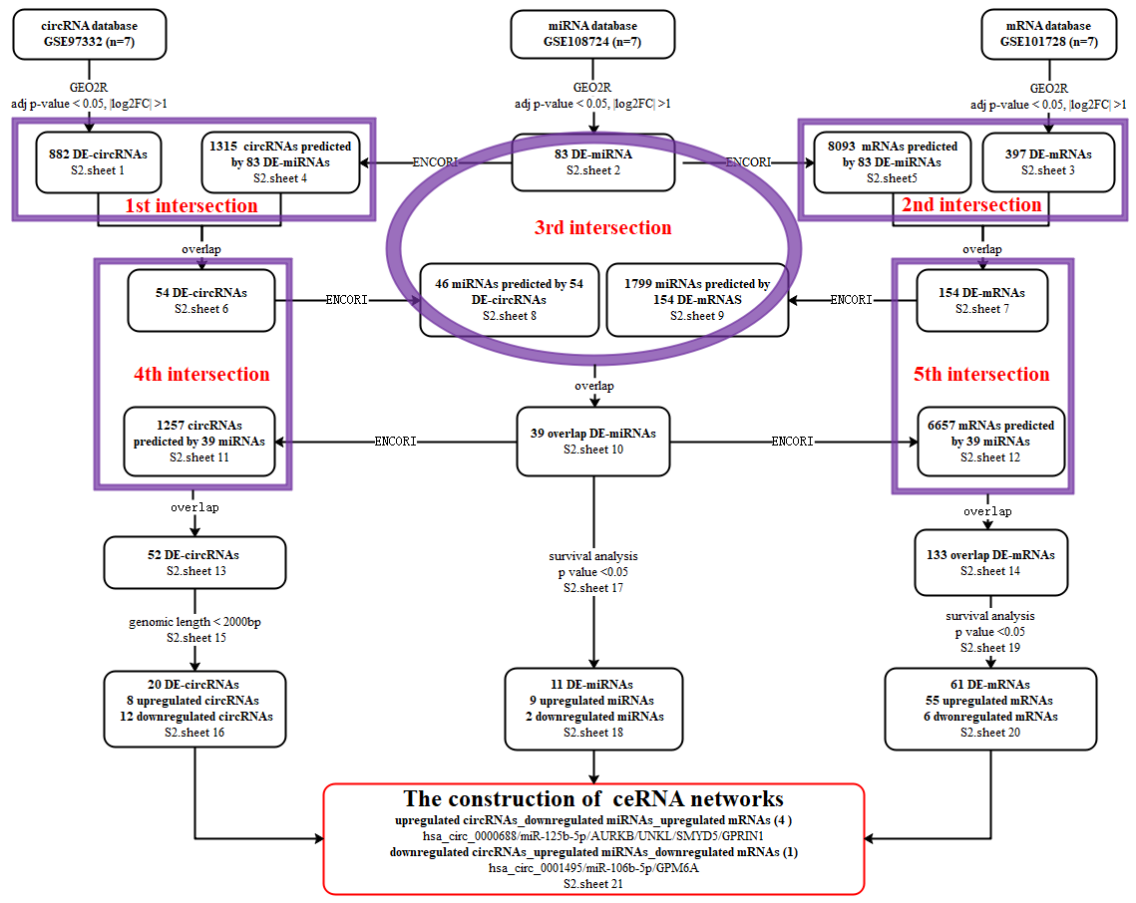
Corrected original Figure 1A.

**Figure B:**
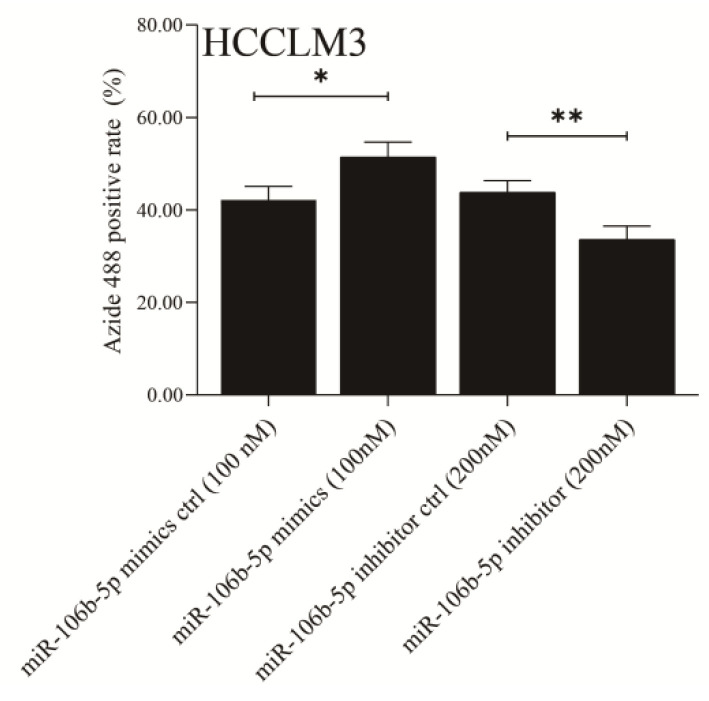
Corrected original Figure 3F.

**Figure C:**
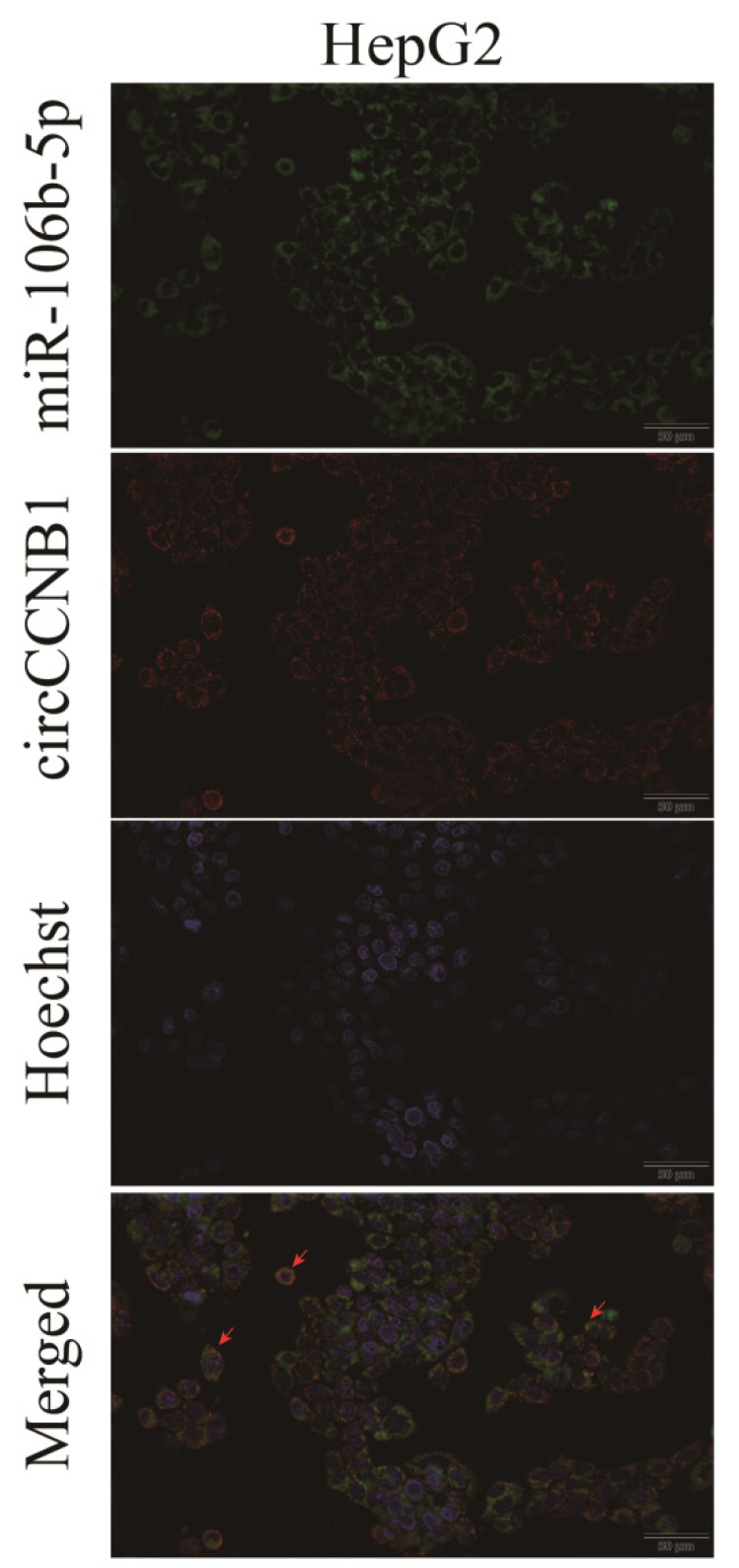
Corrected original Figure 5E.

**Figure D:**
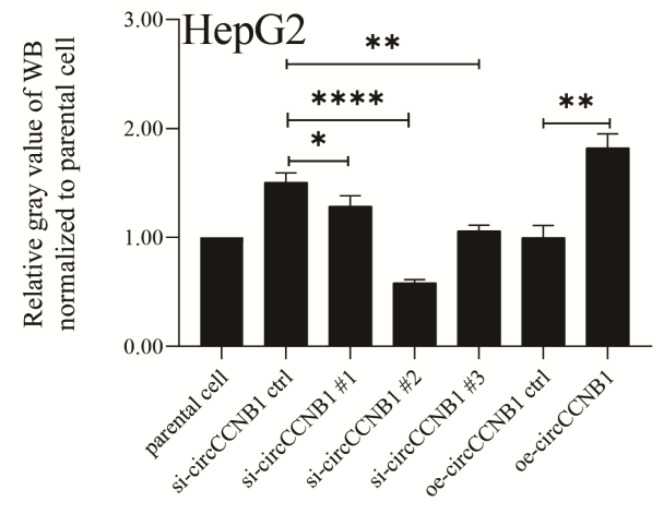
Corrected original Figure S4E.

