# Supplementary material for: CircCCNB1 silencing acting as a miR-106b-5p sponge inhibited GPM6A expression to promote HCC progression by enhancing DYNC1I1 expression and activating the AKT/ERK signaling pathway
Source: Int J Biol Sci. 2022 Jan 1;18(2):637–51. doi: 10.7150/ijbs.66915 (PMC8741844; doi:10.7150/ijbs.66915)
Supplement: Supplementary file 1 — Supplementary figures and table 1. [file ijbsv18p0637s1.pdf]

Supplementary Figure S1. The analysis of functional enrichment, expression differences, and interaction of ceRNAs in HCC based on biological analysis.

The analysis of biological process (A), cellular component (B), molecular function (C), and KEGG pathway (D) was performed for 61 DE-mRNAs using DAVID online software. GPM6A expression based on sample types (E), individual cancer stages (F), tumor grades (G), nodal metastasis status (H), and histological subtypes (I) analyzed by the UALCAN LIHC database. (J). Low GPM6A expression (brown line) was significantly correlated with poor overall survival in HCC (P-value = 0.037, Hazard ratio = 0.69) using online K-M survival analysis of ENCORI. (K). The expression level of miR-106b-5p in HCC cancer tissues is much higher than that of matched para-carcinoma tissues based on ENCORI online pan-cancer analysis. (L). High miR-106b-5p expression (green line) was notably correlated with poor survival in HCC (P-value = 0.019, Hazard ratio = 1.52) using online K-M survival analysis of ENCORI. (M). The expression of GPM6A was negatively correlated with miR-106b-5p expression (r = -0.251 and P-value = 1.01E-06) in HCC clinical tissue samples analyzed using co-expression online tool of ENCORI.

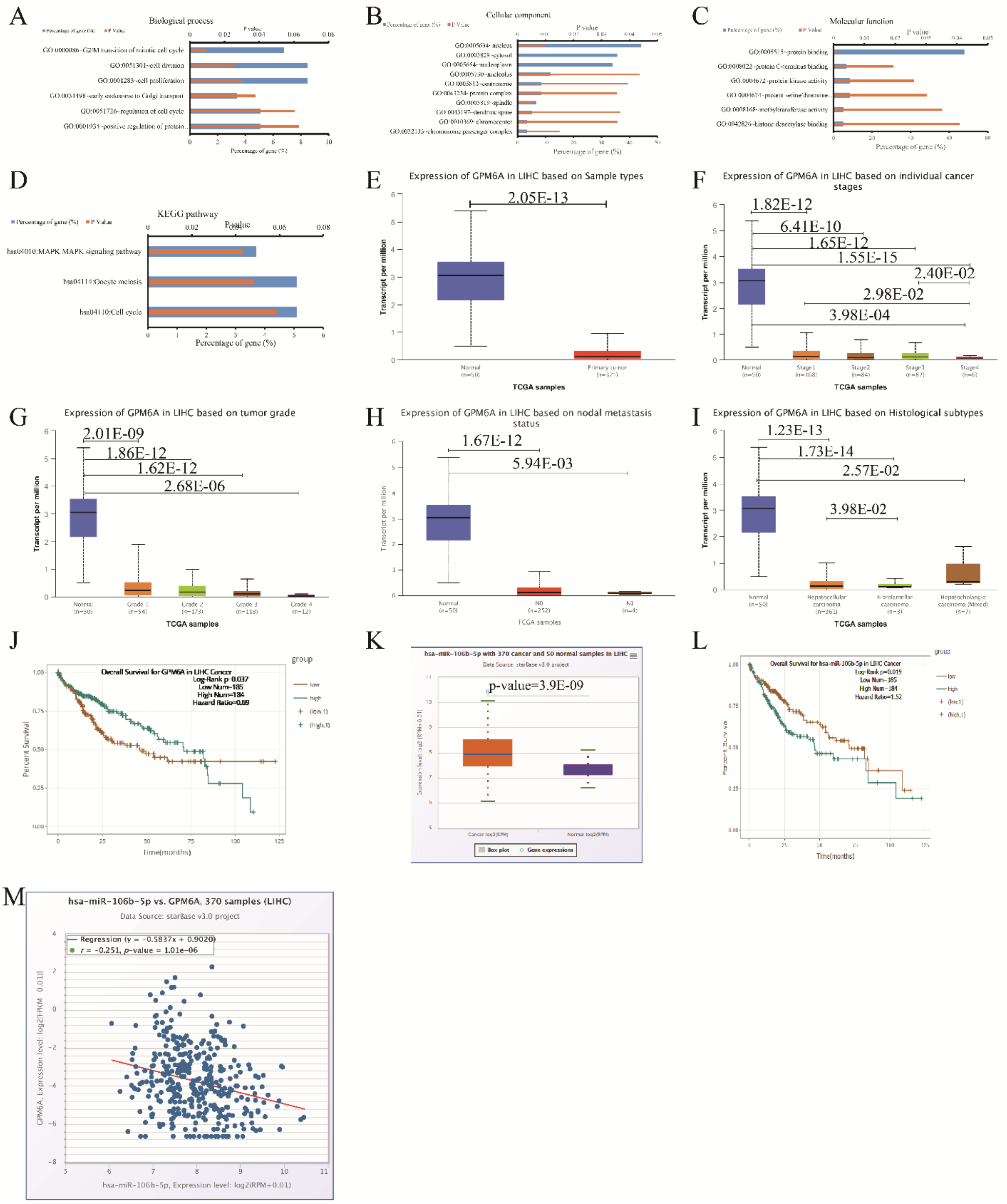
A
Biological process
B
Cellular component
C
Molecular function
D
KEGG pathway
hsa04010:MAPK MAPK signaling pathway
hsa04114:Oocyte meiosis
hsa04110:Cell cycle
Percentage of gene (%)
E
Expression of GPM6A in LIHC based on Sample types
2.05E-13
Transcript per million
TCGA samples
F
Expression of GPM6A in LIHC based on individual cancer stages
1.82E-12
6.41E-10
1.65E-12
1.55E-15
2.40E-02
2.98E-02
3.98E-04
G
Expression of GPM6A in LIHC based on tumor grade
2.01E-09
1.86E-12
1.62E-12
2.68E-06
H
Expression of GPM6A in LIHC based on nodal metastasis status
1.67E-12
5.94E-03
I
Expression of GPM6A in LIHC based on Histological subtypes
1.23E-13
1.73E-14
2.57E-02
3.98E-02
J
Overall Survival for GPM6A in LIHC Cancer
Percent Survival
Time(months)
K
p-value=3.9E-09
L
Time(months)
M
hsa-miR-106b-5p vs. GPM6A, 370 samples (LIHC)
Data Source: starBase v3.0 project
Regression (y = -0.5837x + 0.9020)
r = -0.251, p-value = 1.01e-06
hsa-miR-106b-5p, Expression level: log2(RPM+0.01)

Supplementary Figure S2. MiR-106b-5p mimics and inhibitors, respectively, inhibited and promoted the GPM6A expression in HCC cell lines.

(A) The miR-106b-5p expression level was increased after transfection with miR-106b-5p mimics at the final concentrations of 50nM or 100nM in HepG2 and HCCLM3 cells. The GPM6A relative expression was reversely regulated by up-regulated and down-regulated miR-106b-5p at final concentrations of 100nM or 200nM in HepG2 and HCCLM3 cells detected by qRT-PCR (B) and WB (C). (D, E) Quantification from C. One-way ANOVA analysis was used to compare miR-106b-5p and GPM6A expression between multiple groups. The two independent t-tests were used to compare the difference between any two groups. ns represents not significant, **P*<0.05, ***P*<0.01, ****P*<0.001, *****P*<0.0001.

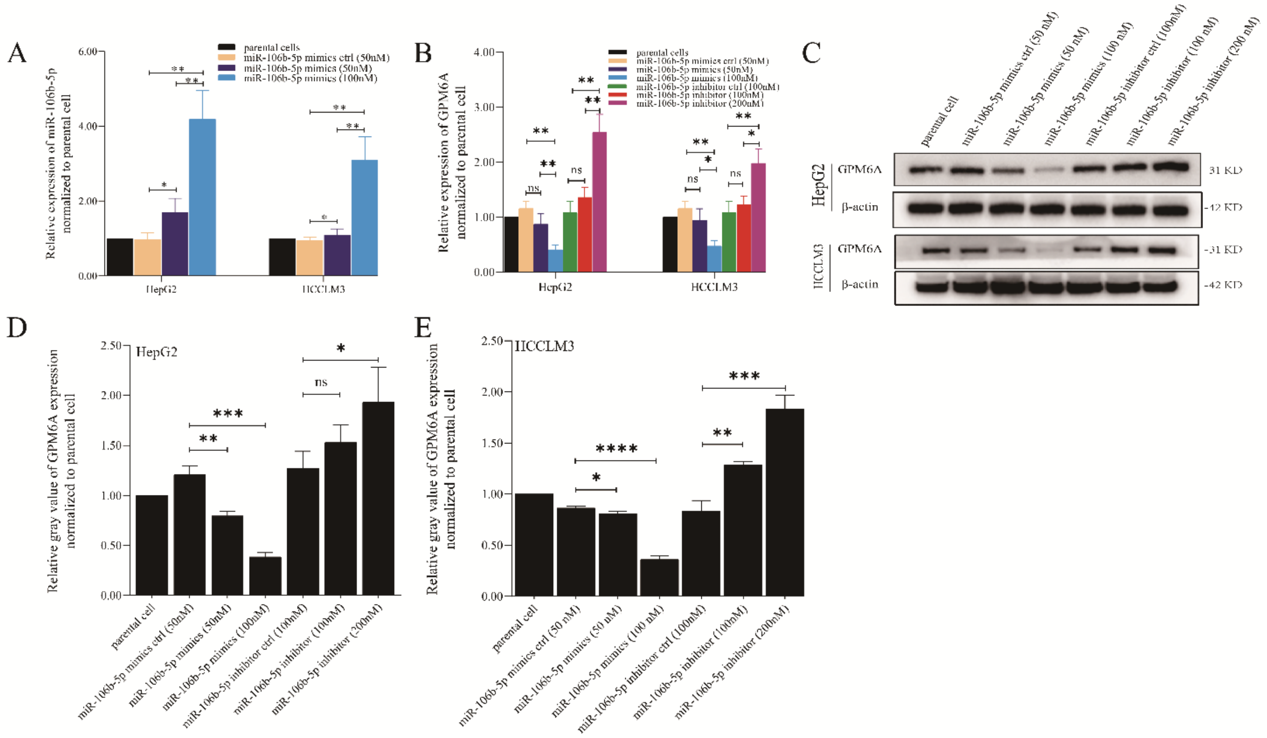

Supplementary Figure S3. MiR-106b-5p mimics and inhibitors, respectively, inhibited and promoted the ability of migration and invasion in HCC cell lines.

The migration (A) and invasion (D) ability of HCC cells were strengthened and suppressed, respectively, by the transfection of miR-106b-5p mimics and inhibitors. (B, C) Quantification from A. (E, F) Quantification from D. The two independent t-tests were used to compare the difference between any two groups. ***P*<0.01, ****P*<0.001, *****P*<0.0001.

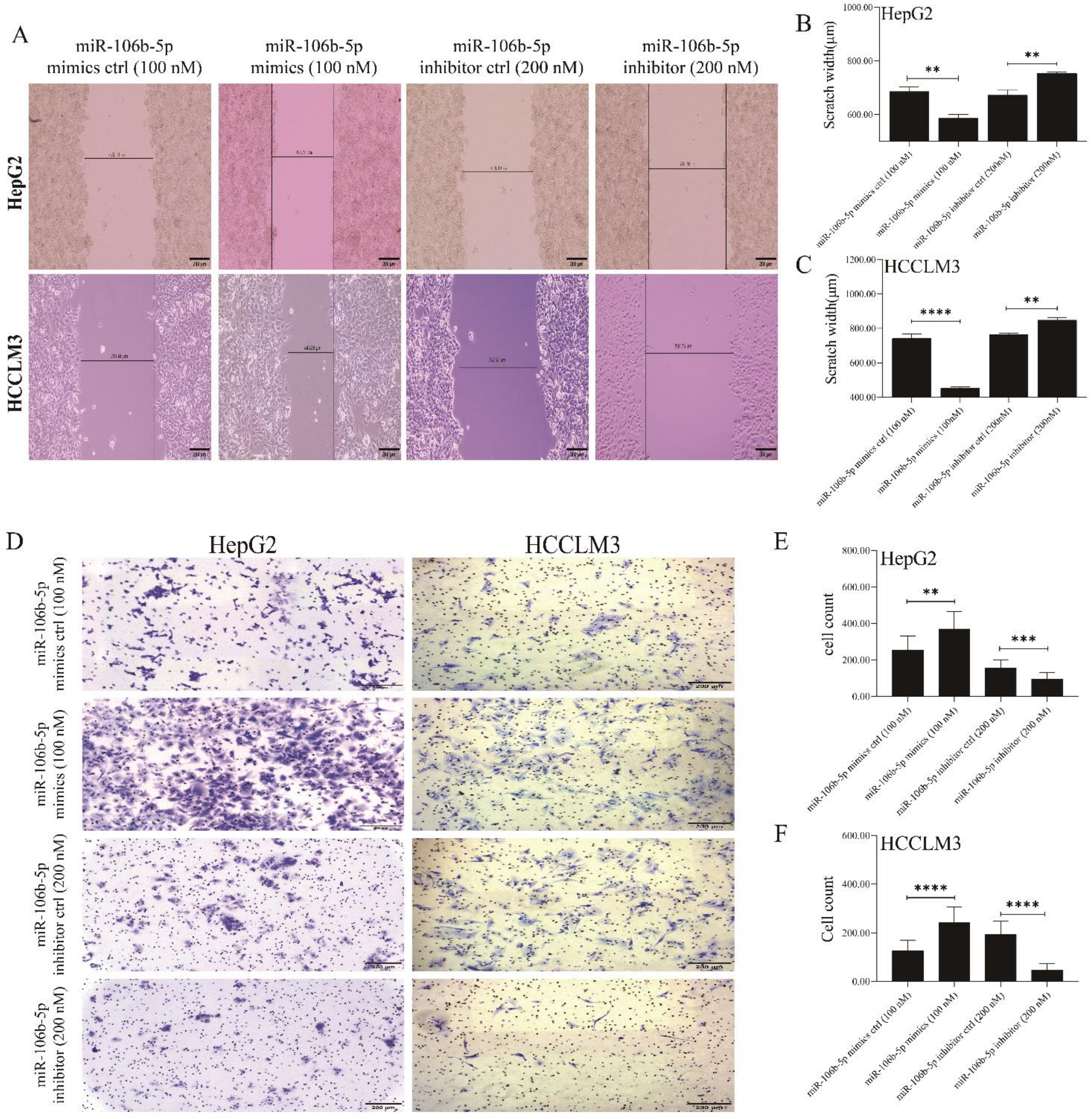

Supplementary Figure S4. Silent and overexpressed circCCNB1, with no effect on the expression of miR-106b-5p, significantly upregulated and downregulated the expression of GPM6A, respectively.

(A) The efficiency of circCCNB1 silencing and overexpression in HepG2 and HCCLM3 cells was assessed by qPCR. The effect of circCCNB1 silencing and overexpression on the expression of miR-106b-5p (B) and GPM6A (C) in HepG2 and HCCLM3 cells measured by qPCR. (D) The GPM6A protein level of HCC cells with circCCNB1 silencing or overexpression were measured by WB method. (E, F) Quantification from D. Data was derived from the results of three independent repeated experiments (Mean ± SD). The two independent t-tests were utilized to compare the difference between any two groups. ns represents not significant, *P<0.05, **P<0.01, ***P<0.001, ****P<0.0001.

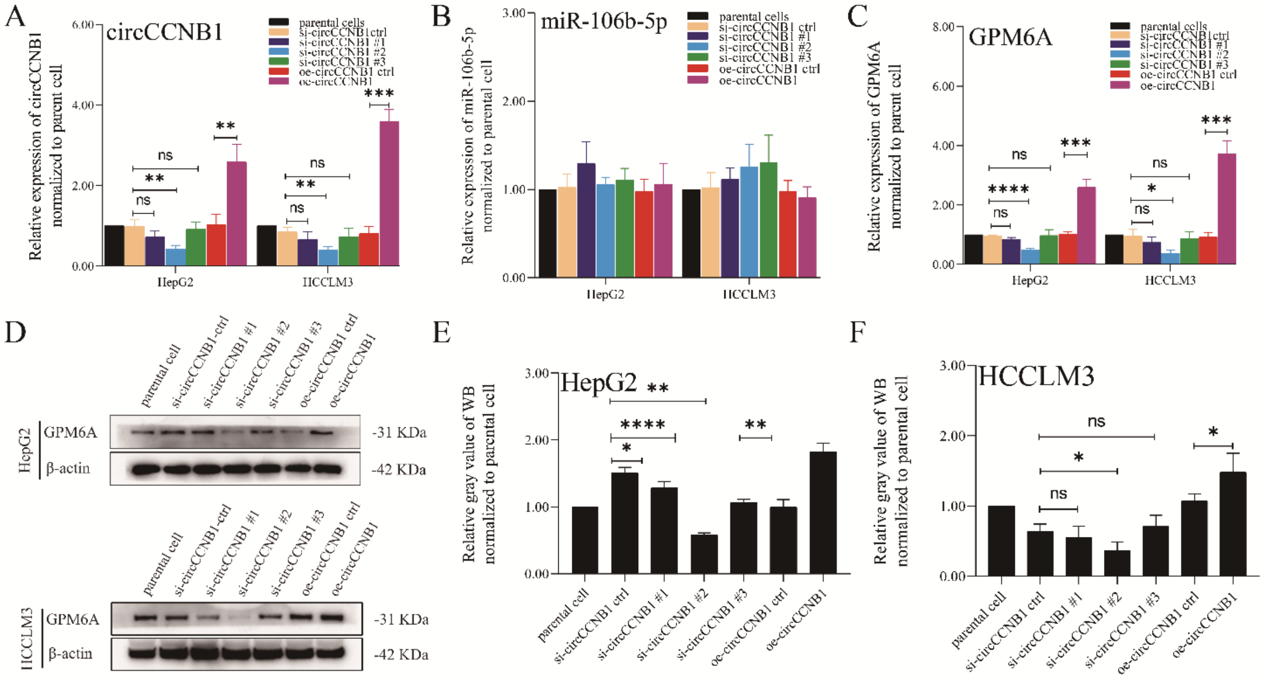

Supplementary Figure S5. Overexpressed DYNC1I1 was closely related to pathogenesis process of HCC. (A-E). DYNC1I1 expression based on sample types (A), individual cancer stages (B), tumor grades (C), nodal metastasis status (D), and histological subtypes (E) analyzed by the UALCAN LIHC database. (F). The expression of GPM6A was negatively correlated with DYNC1I1 expression (r = -0.021 and P-value = 6.58E-01) in HCC clinical tissue samples analyzed using co-expression online tool of ENCORI. (G) High DYNC1I1 expression (green line) was not correlated with poor overall survival of HCC patients (P-value = 0.36) using online K-M survival analysis of ENCORI.

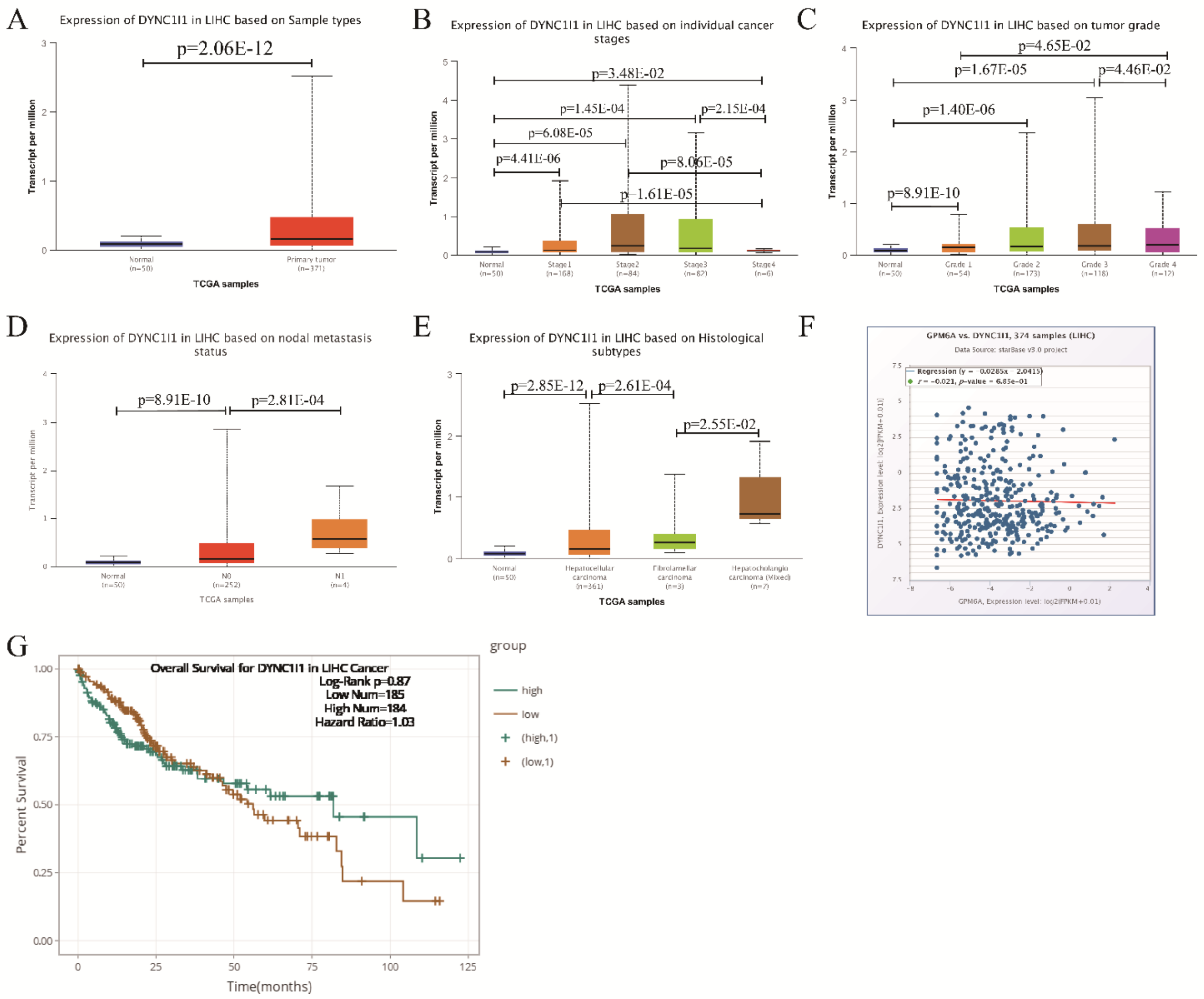

Supplementary files: Table S1. All the primers of RNAs

Genes	Sequences (from 5' to 3')
circCCNB1	Divergent forward primer: CTTCTCAAATTGCAGCAGGAGC
	Divergent reverse primer: ACATGGCAGTGACACCAACCA
GPM6A	Forward primer: TGGCTGGGAGTCACGGCTTT
	Reverse primer: AATGTGGTGTTCCGGCAGATGG
GAPDH	Forward primer: AAGTATGACAACAGCCTCAAG
	Reverse primer: TCCACGATACCAAAGTTGTC
miR-106b-5p	Undisclosed, MQPS0000433
U6	Undisclosed, MQPS0000002
